# RIG-I Signaling Is Critical for Efficient Polyfunctional T Cell Responses during Influenza Virus Infection

**DOI:** 10.1371/journal.ppat.1005754

**Published:** 2016-07-20

**Authors:** Matheswaran Kandasamy, Amol Suryawanshi, Smanla Tundup, Jasmine T. Perez, Mirco Schmolke, Santhakumar Manicassamy, Balaji Manicassamy

**Affiliations:** 1 Department of Microbiology, The University of Chicago, Chicago, Illinois, United States of America; 2 Cancer Immunology, Inflammation, and Tolerance Program, GRU Cancer Center, Georgia Regents University, Augusta, Georgia, United States of America; 3 Department of Microbiology and Molecular Medicine, University of Geneva Medical Faculty, CMU, Geneva, Switzerland; National Institutes of Health, UNITED STATES

## Abstract

Retinoic acid inducible gene-I (RIG-I) is an innate RNA sensor that recognizes the influenza A virus (IAV) RNA genome and activates antiviral host responses. Here, we demonstrate that RIG-I signaling plays a crucial role in restricting IAV tropism and regulating host immune responses. Mice deficient in the RIG-I-MAVS pathway show defects in migratory dendritic cell (DC) activation, viral antigen presentation, and priming of CD8^+^ and CD4^+^ T cell responses during IAV infection. These defects result in decreased frequency of polyfunctional effector T cells and lowered protection against heterologous IAV challenge. In addition, our data show that RIG-I activation is essential for protecting epithelial cells and hematopoietic cells from IAV infection. These diverse effects of RIG-I signaling are likely imparted by the actions of type I interferon (IFN), as addition of exogenous type I IFN is sufficient to overcome the defects in antigen presentation by RIG-I deficient BMDC. Moreover, the *in vivo* T cell defects in RIG-I deficient mice can be overcome by the activation of MDA5 –MAVS via poly I:C treatment. Taken together, these findings demonstrate that RIG-I signaling through MAVS is critical for determining the quality of polyfunctional T cell responses against IAV and for providing protection against subsequent infection from heterologous or novel pandemic IAV strains.

## Author Summary

Retinoic acid inducible gene-I (RIG-I) is a cytosolic RNA sensor that detects influenza virus infection and has been previously shown to be critical for controlling influenza virus replication in cell culture systems. Here, we performed studies in mice with defects in the RIG-I pathway to understand its role in initiating immune responses against influenza virus. Our studies show that RIG-I signaling protects host lung tissue from influenza virus infection and aids in the timely clearance of influenza virus from the lungs. Consistent with this observation, RIG-I deficient mice show defects in type I IFN production and subsequent activation of the adaptive immune response (T cell responses) against influenza viruses. This defect in T cell activation is overcome by exogenous addition of type I IFN to antigen presenting cells (APC) in vitro or by poly I:C treatment of RIG-I deficient mice, demonstrating that RIG-I signaling is critical for type I IFN production and proper activation of adaptive responses against influenza virus infection. Thus, our study suggests that RIG-I signaling is essential for reducing influenza virus disease burden in a timely manner and for providing protection against emerging novel IAV strains.

## Introduction

Influenza A virus (IAV), a member of the *Orthomyxoviridae* family, has a genome composed of eight single-stranded negative sense RNAs, each containing a 5’ triphosphate end (5’-ppp)[1]. IAV infection of a cell is detected by the intracellular innate immune sensor retinoic acid inducible gene-I (RIG-I), which recognizes and binds to the 5’-ppp with double stranded RNA structure found within the panhandle of the IAV genome[2,3]. Upon binding of viral RNA, RIG-I is activated and interacts with the adaptor protein mitochondrial antiviral signaling (MAVS) [also known as interferon-β promoter stimulator-1 (IPS-1) and virus induced signaling adaptor (cardif/VISA)] to elicit antiviral and pro-inflammatory responses through interferon regulatory factor 3 (IRF-3) and nuclear factor κB (NF-κB, respectively[4].

Interferon-β (IFN-β), a member of the type I IFN family, is one of the key antiviral factors induced in response to RIG-I activation[5]. The type I IFN family includes IFN-β and 13 subtypes of IFN-α, which impart their actions by binding to the IFN-α receptor (IFNAR) and invoking a cascade of signaling events that lead to the transcriptional upregulation of more than 300 genes, collectively referred to as interferon stimulated genes (ISGs) [6]. ISGs such as myxovirus resistance gene 1 (Mx1), double stranded RNA activated protein kinase R (PKR), the oligoadenylate synthetase/ribonuclease system (OAS/RNAse L) etc., directly participate in restricting viral replication. In addition to direct antiviral action, type I IFN can modulate the biological function of different cell types of hematopoietic origin [7]. Type I IFN can enhance DC function by upregulating the expression of major histocompatibility complexes (MHC) and co-stimulatory molecules, and directly providing the signal for effector T cell differentiation[8,9]. Moreover, type I IFN can increase the expression of granzyme B (GrB) and enhance the cytolytic activity of CD8^+^ T cells[10]. In concurrence with this observation, IFNAR knock-out mice exhibited increased mortality and morbidity with higher viral loads in the lungs after IAV infection[11]. In addition to IFN-β, proinflammatory cytokines and chemokines induced by activation of the RIG-I pathway participate in the recruitment of immune cells that restrict viral replication and clear virus infected cells.

Previous studies investigating the role of RIG-I signaling in mediating protection against IAV have been performed utilizing mice deficient in the adaptor protein MAVS (MAVS^-/-^) [12,13]. Analysis of T cell responses using peptide-MHC-I tetramer staining indicated similar frequencies of tetramer positive CD8^+^ T cells from both WT and MAVS^-/-^ mice, suggesting that loss of MAVS does not change the frequencies of antigen specific CD8^+^ T cells or that other compensatory mechanisms in MAVS^-/-^ mice may activate adaptive T cell responses. However, it remains unknown if the RIG-I pathway determines the quality of T cell responses against influenza viruses. Given that RIG-I is the key sensor for recognizing IAV infection in epithelial cells, a major cell type targeted by the virus, and the importance of type I IFN in controlling viral infection and modulating DC-T cell functions, we investigated if the RIG-I-MAVS signaling pathway modulates DC activation and T cell function in RIG-I deficient (RIG-I^-/-^) mice. To our knowledge, this is the first study to investigate host immune responses to IAV infection in RIG-I^-/-^ mice. Here, we demonstrate that RIG-I-MAVS signaling is critical for (1) early protection of cell types from IAV infection, (2) optimal activation and antigen presentation by migratory DC, and (3) efficient priming of polyfunctional T cell responses. As such, mice deficient in RIG-I or MAVS showed delayed viral clearance and recovery from primary IAV infection, and decreased protection against subsequent infection from a heterologous IAV strain. Our studies demonstrate that activation of the RIG-I-MAVS pathway is critical for restriction of IAV tropism and efficient priming of T cells, resulting in timely clearance of virus in the respiratory tract as well as protection against subsequent IAV infections.

## Results

### RIG-I deficient mice show delayed clearance of IAV

**Fig 1:**
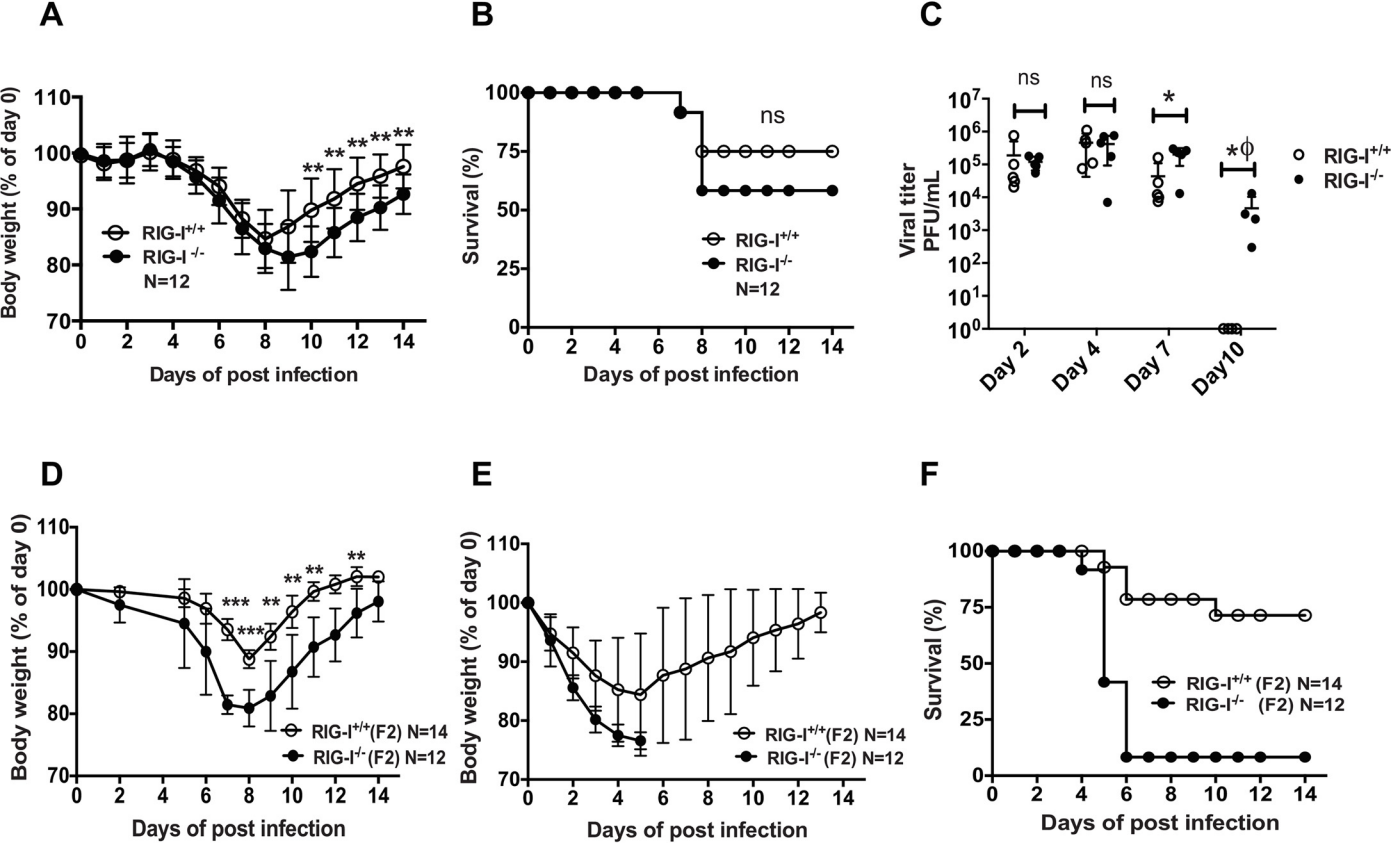
RIG-I deficient mice demonstrate decreased protection against heterologous IAV challenge. (A-B) RIG-I^+/+^ and RIG-I^-/-^ mice were infected with 50 PFU of PR8 and monitored for body weight and survival for 14 days. (A) Percentage of body weight loss after PR8 infection (n = 12 in each group). (B) Survival curve comparing RIG-I^+/+^ and RIG-I^-/-^ mice (n = 12 in each group). (C) Viral titers in the lungs. Viral loads in the lung homogenates were measured by plaque assay. The limit of detection for plaque assay was 10 PFU/ml. (D-F) RIG-I^+/+^ (N = 14) and RIG-I^-/-^ mice (N = 12) were infected with 100 PFU of X-31 and body weight was monitored for 14days. On day 28 post infection, mice were challenged with lethal dose of PR8 (10^6^ PFU) and body weight loss and survival were monitored (D) Percentage of body weight loss after primary infection with X-31. (E) Percentage of body weight loss and (F) Survival curve comparing RIG-I^+/+^ and RIG-I^-/-^ mice after challenge with PR8. All the experiments were independently repeated twice. * Denotes statistical significance at p<0.05. ϕ denotes statistical significance at p<0.05 in Fisher’s exact test, *** denotes statistical significance at p<0.001 and **** denotes statistical significance at p<0.001

Deletion of RIG-I has previously been demonstrated to be embryonically lethal in standard laboratory mice strains such as C57BL6 and BALB/c [5]. Interestingly, in the ICR background, RIG-I deficient mice are viable and develop to adulthood. To investigate the role of RIG-I in mediating protection against IAV infection, mice with functional RIG-I (RIG-I^+/+^) or deficient in RIG-I (RIG-I^-/-^) were infected with 100 PFU of the mouse adapted PR8 strain of IAV (A/Puerto Rico/8/1934; H1N1) and monitored for body weight loss and survival (Fig 1A and 1B) [5]. Despite no difference in the survival between RIG-I^+/+^ and RIG-I^-/-^ mice, delayed recovery of body weight was observed in RIG-I^-/-^ mice (Fig 1B). Since recovery of body weight in infected mice is one of the clinical signs of IAV clearance from the lungs, we examined if the prolonged morbidity in RIG-I^-/-^ mice was due to delayed clearance of IAV. As shown in Fig 1C, we noted similar levels of viral loads in the lungs of RIG-I^+/+^ and RIG-I^-/-^ mice at early times post-infection (pi) (day 2 pi and day 4 pi). Interestingly, on day 7 pi, RIG-I^-/-^ mice showed higher viral loads in the lungs as compared to RIG-I^+/+^ mice, and on day 10 pi, virus was cleared from the RIG-I^+/+^ mice but not in RIG-I^-/-^ mice (Fig 1C). Taken together, these results indicate that RIG-I is critical for the timely clearance of IAV from the lungs.

Previous studies demonstrate that T cell responses generated from primary IAV infection can provide protection against challenge from a heterologous IAV strain carrying the hemagglutinin (HA) and neuraminidase (NA) genes belonging to a different IAV subtype [14,15]. Thus, we investigated if lack of RIG-I affects protection against a heterologous IAV strain. X-31 is a reassortant IAV strain that carries the HA/NA segments from the HK68 strain (A/Hong Kong/1/1968; H3N2) and the remaining internal segments from PR8. RIG-I^-/-^ and RIG-I^+/+^ littermate controls were infected with the X-31 (H3N2) strain at a sublethal dose of 300 PFU and monitored for 14 days (Fig 1D). RIG-I^-/-^ mice infected with X-31 showed delayed recovery of body weight as compared to the RIG-I^+/+^ littermates. At day 14 post-infection, mice from both groups regained their original body weight (day 0). On day 28 post-infection, both groups of mice were challenged with a lethal dose of PR8 (heterologous strain; dose 10^6^ PFU) and monitored for weight loss and survival (Fig 1E and 1F). Interestingly, RIG-I^-/-^ mice showed decreased protection from PR8 challenge with 1/12 mice surviving the infection (8% survival), whereas 10/14 mice in the RIG-I^+/+^ group survived PR8 challenge (71% survival). Taken together, these results indicate that RIG-I is essential for protection against heterologous IAV strains.

### RIG-I^-/-^ mice show decreased polyfunctional T cell responses against IAV

**Fig 2:**
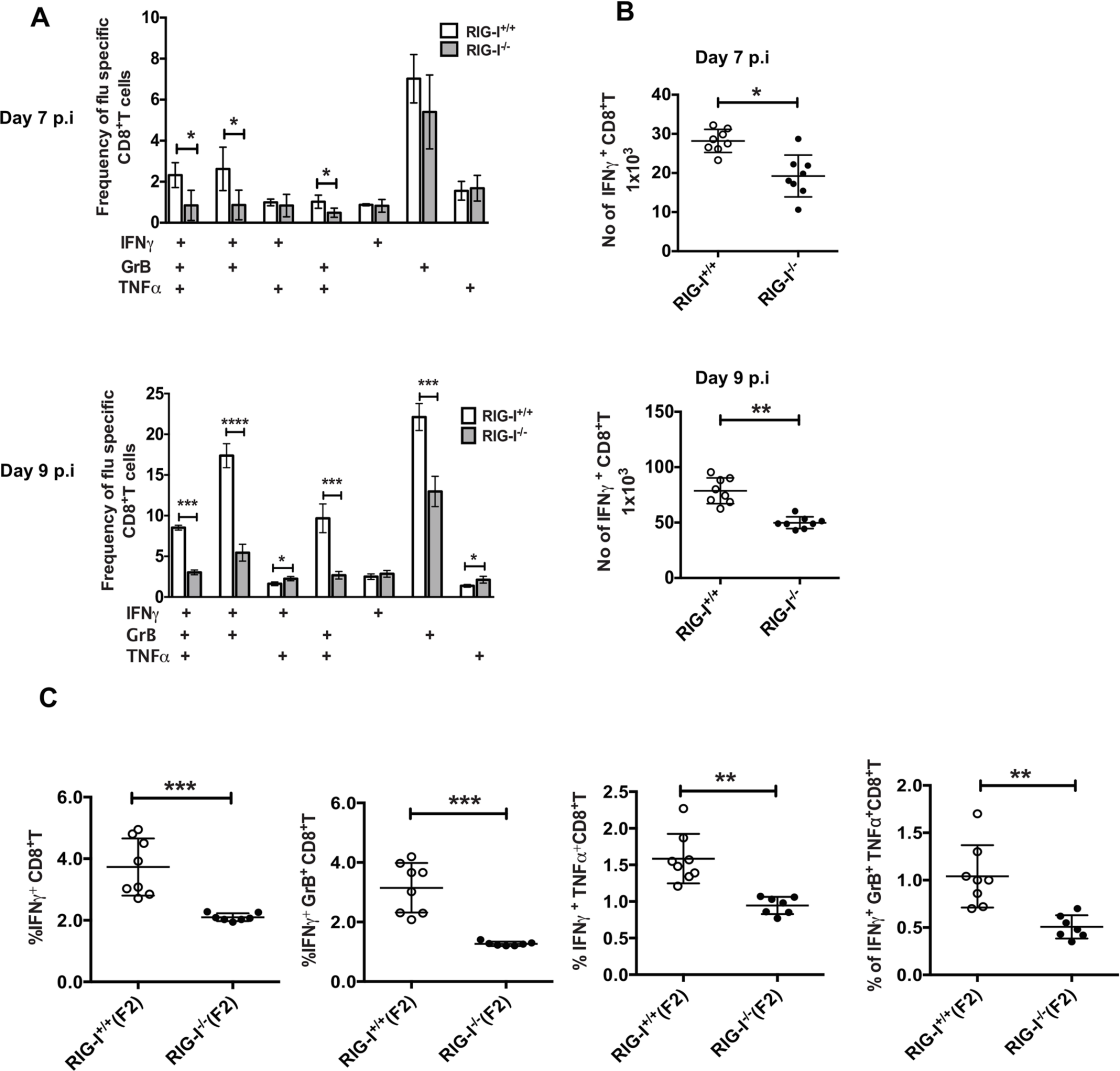
RIG-I deficient mice show impaired T cell response against IAV infection. T cells were isolated from either RIG-I^+/+^ or RIG-I^-/-^ mice on days 7 and 9 post-infection and co-cultured with infected BMDC isolated from RIG-I^+/+^ mice. The frequencies of IFNγ, TNFα and Granzyme B producing CD8^+^ T cells were analyzed by flow cytometry. (A) Bar graphs showing the frequencies of single or polyfunctional CD8^+^T cells on days 7 (upper panel) or day 9 (lower panel) after PR8 infection. (B) Absolute number of IFNγ+ CD8+T cells in lungs on day 7 (upper panel) or day 9 (lower panel) post infection with PR8. (C) Comparison of frequencies of IFNγ, TNFα and GrB producing CD8^+^ T cells between RIG-I^+/+^ and RIG-I^-/-^ littermates. The results shown are a representative of three independent experiments with similar results (n = 8–10 mice/group). The values are expressed as mean ± SEM. * Denotes statistical significance at p<0.05., *** denotes statistical significance at p<0.001 and **** denotes statistical significance at p<0.0001.

As primary IAV infection and protection against heterologous IAV strains are mediated by actions of T cells, we investigated if there are any defects in CD8^+^ T cell responses in RIG-I^-/-^ mice. Unfortunately, the resources for performing standard tetramer staining to analyze T cell responses in the ICR background are limited, as the IAV specific MHC-I epitopes are still undetermined. Hence, we measured polyclonal T cell responses by analyzing the expression of IFN gamma (IFNγ), tumor necrosis factor alpha (TNFα), and Granzyme B (GrB). T cells were isolated on day 7 or 9 pi from the lungs of RIG-I^+/+^ and RIG-I^-/-^ mice infected with PR8 and co-cultured with RIG-I^+/+^ BMDC previously infected with PR8. The expression of IFNγ, TNFα and GrB in CD8^+^T cells was measured by intracellular staining followed by flow cytometric analysis (Fig 2A and S1A Fig). CD8^+^ T cells from RIG-I^-/-^ mice showed significantly decreased levels of IFNγ, TNFα, and GrB as compared to CD8^+^ T cells from RIG-I^+/+^ mice (Fig 2A). Importantly, the frequencies of polyfunctional CD8^+^ T cells (triple producers = IFNγ^+^ GrB^+^ TNFα^+^, double producers = IFNγ^+^ GrB^+^ or GrB^+^ TNFα^+^) were lower in RIG-I^-/-^ mice (Fig 2A). Similarly, the polyfunctional CD4^+^ T cell responses in RIG-I^-/-^ mice were lower as compared to RIG-I^+/+^ mice (S2 Fig). It should be noted that co-culture of naïve RIG-I^+/+^ BMDC (no PR8 infection) with T cells from infected mice resulted in non-specific production of GrB^+^ T cells. However, IFNγ production and polyfunctional cytokine production were observed only in the presence of viral antigen (S1A Fig). Next, we investigated if the absolute numbers of IFNγ^+^ CD8+ T cells were different between these two groups, and observed lowered numbers of IFNγ^+^ CD8+ T cells in lung of RIG-I^-/-^ mice as compared to RIG-I^+/+^ mice on days 7 and 9 pi (Fig 2B). These data demonstrate that lack of RIG-I signaling results in lowered numbers of IAV specific CD8^+^ T cells.

To further demonstrate that decreased CD8^+^ T cell responses were due to a defect in RIG-I, as well as to rule out other genetic discrepancies contributing to the difference in T cell responses, we examined the T cell responses in RIG-I^+/+^ and RIG-I^-/-^ littermates generated from crossing mice heterozygous for RIG-I (RIG-I^+/-^) by measuring the production of IFNγ, TNFα, and GrB (Fig 2C and S1B Fig). We observed lowered polyfunctional CD8^+^ T cell responses in RIG-I^-/-^ as compared to RIG-I^+/+^ littermates, as determined by the expression of IFNγ, TNFα, and GrB (Fig 2C). Collectively, these results demonstrate that RIG-I is critical for the efficient activation of polyfunctional T cell responses against IAV infection.

Poor T cell responses can be mainly attributed to either intrinsic defects in the T cell population or impaired antigen presentation by RIG-I^-/-^ antigen presenting cells (APC). To exclude the possibility of an intrinsic T cell defect, T cells isolated from the lungs or draining lymph nodes of RIG-I^+/+^ and RIG-I^-/-^ mice were stimulated with anti-CD3/CD28 antibodies or with PMA/ionomycin and analyzed for proliferation and CD69 upregulation or IFNγ production, respectively (S3 Fig). Upon anti-CD3/CD28 stimulation, T cells from both RIG-I^+/+^ and RIG-I^-/-^ groups showed similar levels of proliferation and upregulation of CD69 expression (S3A–S3D Fig). Similarly, treatment with PMA/ionomycin lead to an increase in IFNγ production as compared to unstimulated cells (S3E and S3F Fig). In addition, we did not observe any significant differences in the levels of IFNγ production between T cells isolated from these two groups of mice. This suggests that the lowered levels of polyclonal T cell responses observed in BMDC-T cell culture were not due to any intrinsic defects in RIG-I^-/-^ T cells (Fig 2).

### RIG-I^-/-^ BMDCs are unable to stimulate T cells

**Fig 3:**
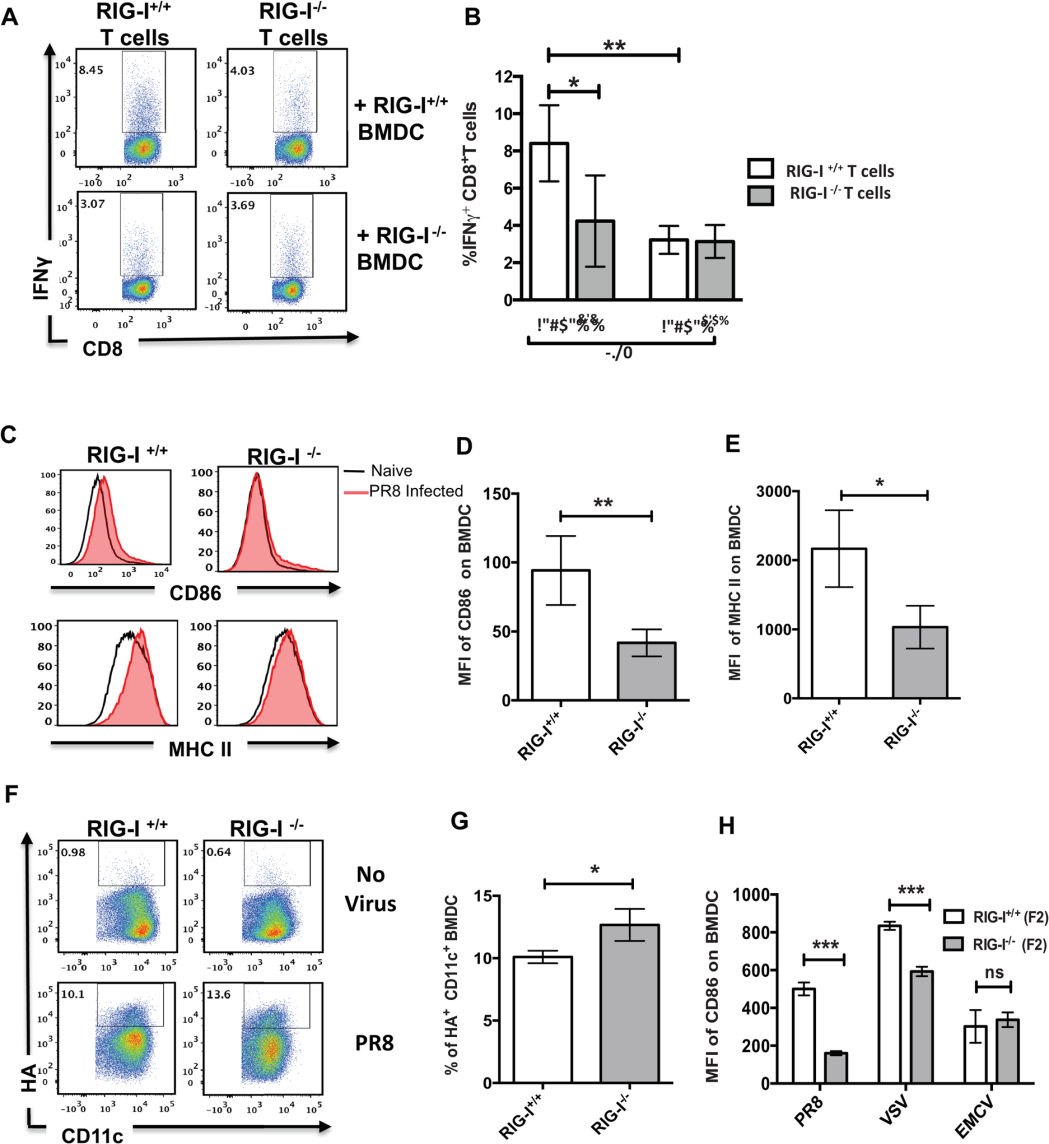
RIG-I deficient BMDC are inefficient in antigen presentation to T cells. T cells were isolated from PR8 infected RIG-I^+/+^ and RIG-I^-/-^ mice and co-cultured with BMDC generated from RIG-I^+/+^ and RIG-I^-/-^ mice or vice versa. The levels of IFNγ production in CD8^+^ T cells were determined by flow cytometry (n = 8/group). (A) Representative dot plots showing IFNγ production. (B) Quantification of panel A. (C) Histograms showing the expression of costimulatory molecules CD86 and MHC-II on RIG-I^+/+^ or RIG-I^-/-^ mice. BMDC were either mock infected (clear) or with IAV at a MOI of 0.5 (shaded). (D) Quantification of the mean fluorescent intensity (MFI) of CD86 upregulation on PR8 infected BMDC with relative to naïve control. (E) Quantification of MFI of MHC II upregulation on PR8 infected BMDC with relative to naïve control. (F-G) Susceptibility of RIG-I^+/+^ or RIG-I^-/-^ BMDC to IAV infection. BMDC were infected overnight with an MOI of 0.5 and the infected population was identified by staining for viral hemagglutinin (HA) protein. (F) Representative dot plots showing HA+ BMDC. (G) Quantification of the frequencies of HA+ BMDC. (H) Comparison of MFI of CD86 upregulation on BMDC infected with different viruses. The upregulation of CD86 expression in infected BMDC was calculated by subtracting the MFI with MFI of naïve state. The data shown in panels A-B is from a representative experiment performed with n = 8 mice/group. The experiments were repeated twice. Data shown in panels C-H is a representative of 2 independent experiments done in triplicates. The values are expressed as mean ± SEM. * Denotes statistical significance at p<0.05 and ** denotes statistical significance at p<0.01.

Next, we examined whether the lowered polyfunctional T cell responses observed in RIG-I^-/-^ mice were due to inefficient antigen presentation by DC. To study this, we evaluated the ability of RIG-I^+/+^ and RIG-I^-/-^ BMDC to present antigens to T cells isolated from either RIG-I^+/+^ or RIG-I^-/-^ mice infected with PR8 (Fig 3). Interestingly, in co-cultures with RIG-I^-/-^ BMDC, we observed lower frequencies of IFNγ+ T cells for both groups of mice, as compared to co-cultures with RIG-I^+/+^ BMDC and RIG-I^+/+^ T cells (Fig 3A and 3B). This indicates that RIG-I^-/-^ BMDC are unable to restimulate T cells from infected mice. As described in Fig 2, there were fewer IFNγ+ RIG-I^-/-^ T cells in the co-cultures of RIG-I^+/+^ BMDC due to inefficient activation T cells *in vivo* in the RIG-I^-/-^ mice. Furthermore, we observed inefficient activation of RIG-I^-/-^ BMDC, as evidenced by lower levels of CD86 and MHC-II expression when compared to RIG-I^+/+^ BMDC (Fig 3C and 3D). Despite less activation, PR8 infection levels were higher in RIG-I^-/-^ BMDC as compared to RIG-I^+/+^ BMDC (13.6% vs 10% HA^+^ cells), suggesting that RIG-I^-/-^ BMDC have an increased susceptibility to IAV infection (Fig 3F and 3G). To understand the molecular basis for the increased susceptibility of RIG-I^-/-^ BMDC to PR8 infection, we profiled the expression of antiviral genes and observed lowered levels of IFN-β, antiviral ISGs, and inflammatory cytokines in PR8 infected RIG-I^-/-^ BMDC as compared to RIG-I^+/+^ BMDC (S4 Fig). These data demonstrate that RIG-I signaling is critical for BMDC activation as well as for efficient stimulation of T cells. Next, we investigated if RIG-I^-/-^ BMDC respond to signaling via the MDA5 –MAVS pathway by performing infections with Encephalomyocarditis virus (EMCV). RIG-I^-/-^ BMDC infected with EMCV showed upregulation of the activation marker CD86 at levels similar to RIG-I^+/+^ BMDC, suggesting that RIG-I^-/-^ BMDC do not have any defect in responding to viral infection via MDA5 (Fig 3H). In contrast, vesicular stomatis virus infection (VSV), which is sensed via RIG-I, showed decreased activation of RIG-I^-/-^ BMDC. Taken together, our BMDC experiments show that RIG-I is essential for (1) restriction of IAV replication, (2) proper activation of DC, and (3) efficient antigen presentation to T cells *ex vivo*.

### *In vivo* RIG-I signaling is critical for protecting respiratory cell subsets from IAV infection

As we observed increased susceptibility of RIG-I^-/-^ BMDC to PR8 infection (Fig 3F and 3G), we next investigated whether RIG-I signaling is critical for protecting cells from IAV infection (gating strategy in S5 and S6 Figs). Surface staining analysis for viral hemagglutinin (HA) protein showed an increased number of HA^+^ migratory DC (CD103^+^ DC, CD11b^+^ DC) and HA^+^ macrophages (alveolar and interstitial) in the lungs of infected RIG-I^-/-^ mice as compared to infected RIG-I^+/+^ mice. In addition, the numbers of infected non-hematopoietic (CD45^-^) cells, most likely epithelial cells, were higher in RIG-I^-/-^ mice as well. To determine if the increased susceptibilities of multiple cell types to IAV infection was due to decreased host responses, we performed qPCR analysis of inflammatory genes in the lungs on days 2 and 4 pi (S6 Fig). These results demonstrate that loss of RIG-I signaling results in reduced host antiviral responses, which is necessary for protecting respiratory cell subsets from IAV infection.

### *In vivo* RIG-I signaling is critical for early activation of CD103+ migratory DCs

**Fig 4:**
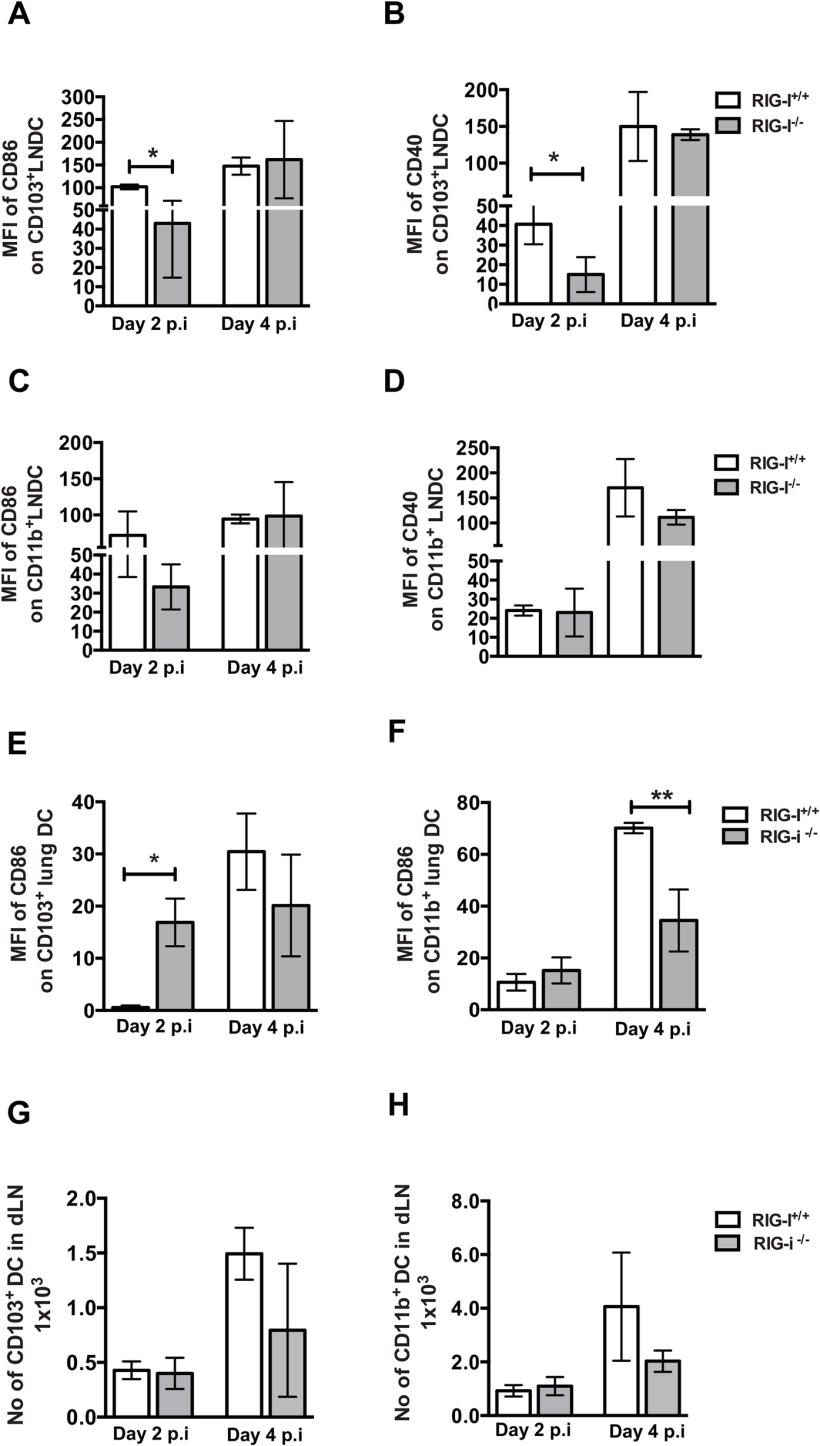
Migratory CD103+ DC in the MLN show decreased expression of CD86 and CD40. RIG-I^+/+^ and RIG-I^-/-^ mice were infected with 50 PFU of PR8 and CD86 expression on migratory DC present in the mediastinal lymph node and lungs were analyzed by flow cytometry. (A-D) Quantification of MFI of CD86 and CD40 upregulation on migratory DC in PR8 infected mice MLN relative to naïve controls. (A, B) CD103^+^ DC and (C,D) CD11b^+^ DC. (E-F) Quantification of MFI of CD86 and CD40 upregulation on migratory DC in PR8 infected mice lungs with relative to naïve mice. (E) CD103^+^ DC and (F) CD11b^+^ DC. (G-H) Absolute numbers of migratory DC in the MLN on day 2 and 4 post infection. (G) CD103^+^ DC and (H) CD11b^+^ DC. Data presented here is the average of three independent experiments (n = 11/group). * Denotes statistical significance at p<0.05 and ** denotes statistical significance at p<0.01

In our *ex vivo* studies, BMDC from RIG-I^-/-^ mice showed lowered levels of CD86 and MHCII upregulation upon infection with IAV (Fig 3D and 3E). Next, we analyzed if RIG-I mediated signaling *in vivo* is critical for migratory DC activation and migration to mediastinal lymph nodes (MLN) during IAV infection. We performed CFSE labeling and tracking studies in RIG-I littermates infected with PR8 (S7 Fig) and observed similar levels of CFSE labeled CD103^+^ and CD11b^+^ DC in the MLN of RIG-I^+/+^ and RIG-I^-/-^ mice, suggesting no significant defects in DC migration. We next examined the activation statuses of different migratory DC populations in the MLN, which are responsible for priming naïve CD8^+^ and CD4^+^ T cells (Fig 4; representative histograms are shown in S8 Fig). Interestingly, CD103^+^ DC in the MLN showed lowered levels of CD86 and CD40 expression in RIG-I^-/-^ mice on day 2 pi as compared to RIG-I^+/+^ mice, whereas there was no significant difference in the levels of CD86 and CD40 expression for CD11b^+^ DC (Fig 4A–4D). However, CD86 expression on CD103^+^ DC was higher in RIG-I^-/-^ lungs on day 2 pi as seen in Fig 4E and 4F. These results indicate that RIG-I signaling is critical for early activation of CD103+ migratory DC present in the MLN, where naïve T cell priming occurs.

### MAVS deficient mice display decreased polyfunctional T cell responses

**Fig 5:**
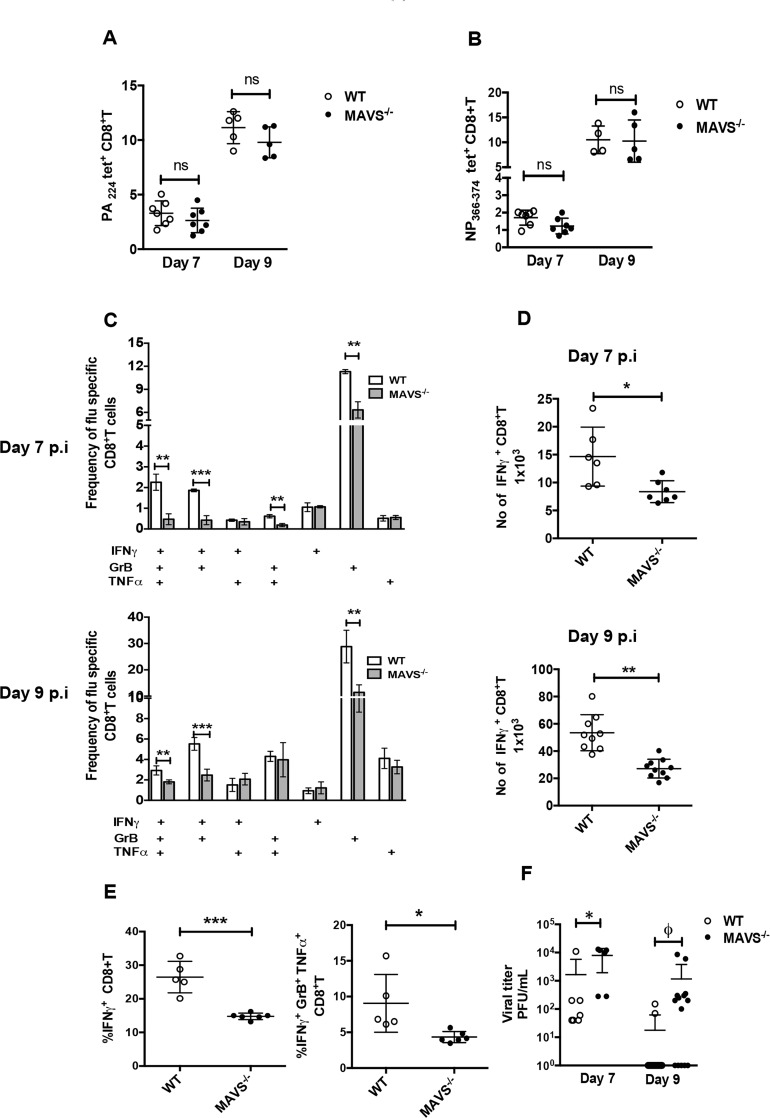
MAVS deficient mice display decreased polyclonal CD8^+^ T cell responses and increased viral loads in the lungs. WT or MAVS^-/-^ mice were infected with 50 PFU of PR8. On day 7 and 9 pi, T cell responses and viral titers in the lungs were determined. (A) Quantification of the frequency of PA and (B) NP specific lung CD8^+^ T cells as determined using tetramers (PA_224_ and NP_366-374_) on day 7 and 9 post-infection. (C) Bar graphs showing the frequencies of single or polyfunctional CD8^+^T cells on days 7 (upper panel) or 9 (lower panel) post infection. (D) Absolute number of IFNγ^+^ CD8+T cells in lungs on day 7 (upper panel) or day 9 (lower panel) post infection. (E) Quantification of the frequencies of IFNγ or IFNγ, GrB and TNFα secreting CD8^+^ T cells in ex vivo stimulation with NP_366-374_ peptide. (F) Viral titers in the lungs on day 7 and 9 post-infection are shown as PFU/ml. The limit of detection for plaque assay was 10 PFU/ml. Data presented panel A-F is a representative of two indepdent experiment performed with n = 5–11 mice/group. The experiments were performed twice independently. * Denotes statistical significance at p<0.05., *** denotes statistical significance at p<0.001 and **** denotes statistical significance at p<0.0001. ϕ denotes statistical significance at p<0.05 in Fisher’s exact test.

To further investigate if RIG-I signaling through MAVS is critical for efficient activation of T cells, we used tetramers specific for IAV proteins PA or NP to evaluate CD8^+^ T cell responses to IAV infection in MAVS^-/-^ mice. Unlike deletion of the RIG-I gene, deletion of MAVS does not affect the viability or development of standard laboratory mice. In corroboration with prior studies, we observed similar frequencies of NP and PA specific tetramer positive CD8^+^ T cells in MAVS^-/-^ and WT counterpart mice (Fig 5A and 5B)[12,13]. However, as evidenced in RIG-I^-/-^ mice in Fig 2, CD8^+^ T cells from MAVS^-/-^ mice expressed lowered levels of IFNγ, GrB, and TNFα (Fig 5C and representative plots are shown in S9 Fig). Importantly, the frequency of polyfunctional CD8^+^ T cells was significantly lower in MAVS^-/-^ mice than in WT controls on days 7 and 9 pi (Fig 5C). It should be noted that co-culture of naïve BMDC with T cells from infected mice resulted in the non-specific production of GrB. IFNγ production and polyfunctionality were observed only in T cells co-cultured with infected BMDC. Similar to our results in RIG-I^-/-^ mice, we observed lowered numbers of IFNγ producing T cells in the lungs of MAVS^-/-^ mice as compared to WT mice, demonstrating that loss of MAVS results in poor T cell responses against IAV (Fig 5D). Next, we examined polyfunctional T cell responses against a dominant IAV epitope by stimulating with NP_366-374_ peptide. The frequency of polyfunctional T cells with specificity to NP_366-374_ were significantly decreased in MAVS^-/-^ mice as compared to WT littermate controls (Fig 5E and S9D Fig). Next, we examined if lowered CD8^+^ T cell responses affect viral clearance in the lungs of MAVS^-/-^ mice. As observed in RIG-I^-/-^ mice (Fig 1C), MAVS^-/-^ mice showed higher viral loads in the lungs on days 7 and 9 pi as compared to WT littermates (Fig 5F). These data are in agreement with our RIG-I^-/-^ mice studies, demonstrating the importance of the RIG-I-MAVS pathway in activation of polyfunctional CD8^+^ T cell responses and timely clearance of IAV in the lungs.

### MAVS deficient mice demonstrate inefficient CD8 and CD4 T cell priming *in vivo*

**Fig 6:**
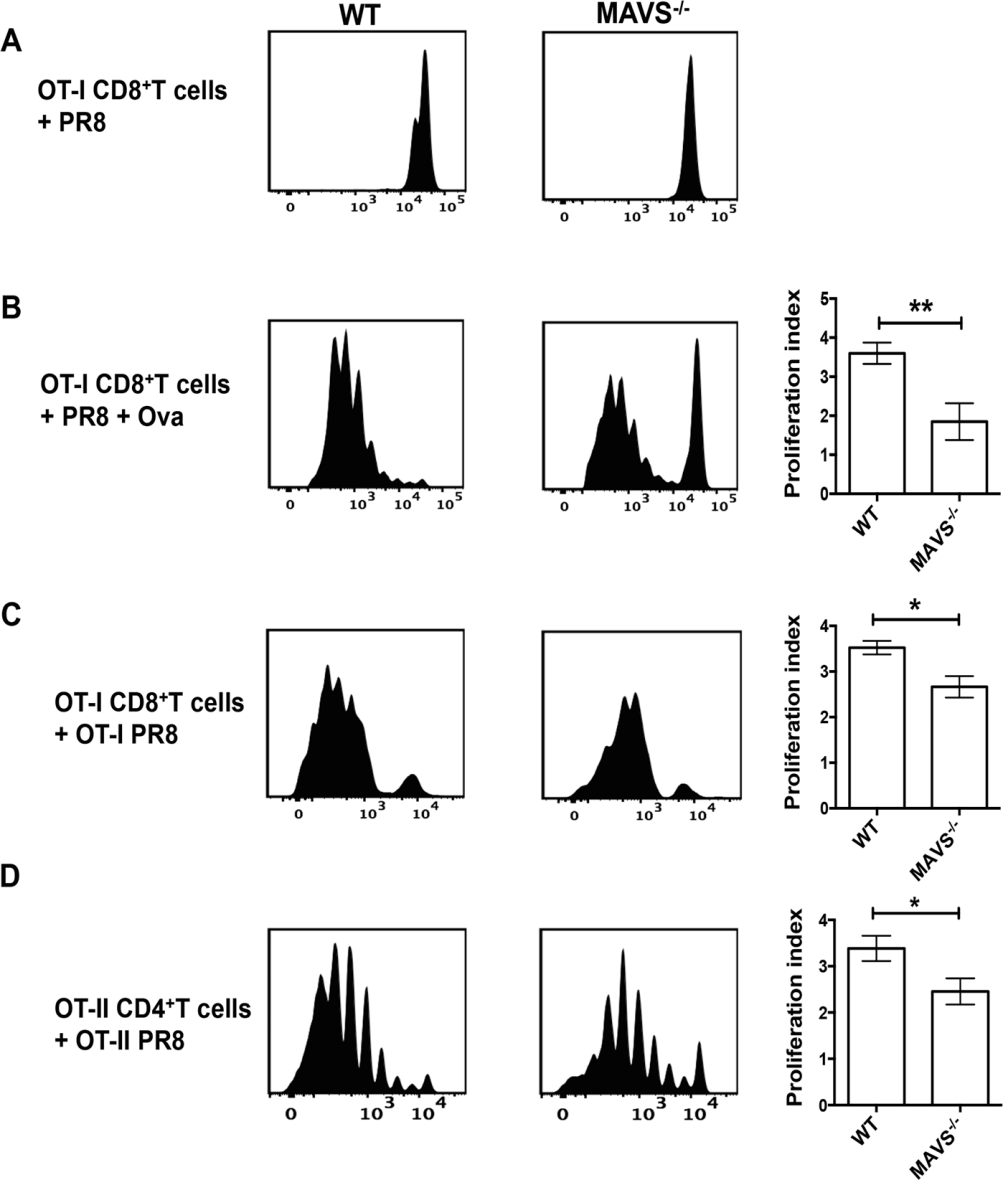
MAVS deficient mice show impaired CD8 and CD4 T cell priming. WT or MAVS^-/-^ mice were adoptively transferred with 2x10^6^ CFSE-labeled OTI CD8^+^ T cells (A-C) or 3x10^6^ CFSE-labeled OTII CD4^+^ T cells (D), and their proliferation were determined on day 3 or 4 post infection with 100PFU PR8. Left-A representative plot showing proliferation of CFSE-labeled T cells. Right-Proliferation index of T cells. (A-C) Proliferation of OT-I cells on day 3 post infection. (A) PR8 alone, (B) PR8 with 60μg of LPS free ovalbumin, and (C) PR8-OTI. (D) WT or MAVS^-/-^ mice were adoptively transferred with 3x10^6^ CFSE-labeled OTII CD4^+^ T cells infected with PR8-OTII in the MLN, on day 4 post-infection. The values are expressed as mean ± SEM. Data presented here is an average of two independent experiments with n = 9 mice/group. * Denotes statistical significance at p<0.05 and ** denotes statistical significance at p<0.01.

Previous studies from our group and others have demonstrated that CD103^+^ migratory DC in the lungs acquire exogenous antigens and prime naïve CD8^+^ T cells via antigen cross-presentation [16,17,18]. Our *ex vivo* studies with BMDC generated from RIG-I^-/-^ mice showed decreased upregulation of CD86/MHCII and inefficient direct antigen presentation to T cells. Thus, we investigated if the observed decrease in polyfunctional T cell responses in MAVS^-/-^ mice was due to inefficient priming of naïve T cells (Fig 6). WT and MAVS^-/-^ mice were adoptively transferred with CFSE labeled naïve OT-I CD8^+^ T-cells followed by intranasal infection with PR8 with or without endotoxin free ovalbumin (Ova) as an exogenous antigen. On day 4 pi, MLN were isolated and the CFSE dilution of OT-I CD8^+^ T cells was analyzed as a measure of cross-priming efficiency. We observed higher levels of OT-I CD8^+^ T cell proliferation in WT mice that received PR8 and Ova as compared to MAVS^-/-^ mice (Fig 6B). Next, we investigated if priming of T cells specific for the epitopes expressed as part of viral proteins is affected in MAVS^-/-^ mice. MAVS^-/-^ and WT littermate controls were adoptively transferred with either OT-I or OT-II T cells and infected with a recombinant PR8 strain carrying Ova epitopes. Interestingly, the proliferation of both OT-I and OT-II cells was lower in MAVS^-/-^ as compared to WT littermate controls (Fig 6C and 6D). Together, these results demonstrate that *in vivo* activation of the RIG-I-MAVS pathway is necessary for efficient antigen presentation and priming of CD8^+^ T cells during IAV infection.

### Exogenous IFN improves antigen presentation and T cell activation by RIG-I deficient BMDC

To investigate if inefficient antigen presentation by RIG-I^-/-^ BMDC was due to a lack of sufficient type I IFN levels, we performed BMDC-T cell co-culture experiments in the presence of exogeneous type I IFN. BMDC derived from RIG-I^+/+^ and RIG-I^-/-^ mice were co-cultured with T cells isolated from RIG-I^+/+^ mice previously infected with PR8. The addition of IFN to BMDC increased the expression of CD86 and MHC II for both RIG-I^+/+^ and RIG-I^-/-^ groups (S10A and S10B Fig). Furthermore, exogenous IFN was sufficient to restore levels of IFNγ producing CD8^+^ T cells re-stimulated by RIG-I^-/-^ BMDC to levels similar to RIG-I^+/+^ BMDC (S10C Fig). These data indicate that the inefficient T cell responses observed in RIG-I^-/-^ mice may be in part due to lower levels of type I IFN in the lungs, impairing migratory DC activation and thus resulting in inefficient T cell responses and delayed viral clearance.

### Poly I:C treatment improves T cell responses in RIG-I deficient mice

**Fig 7:**
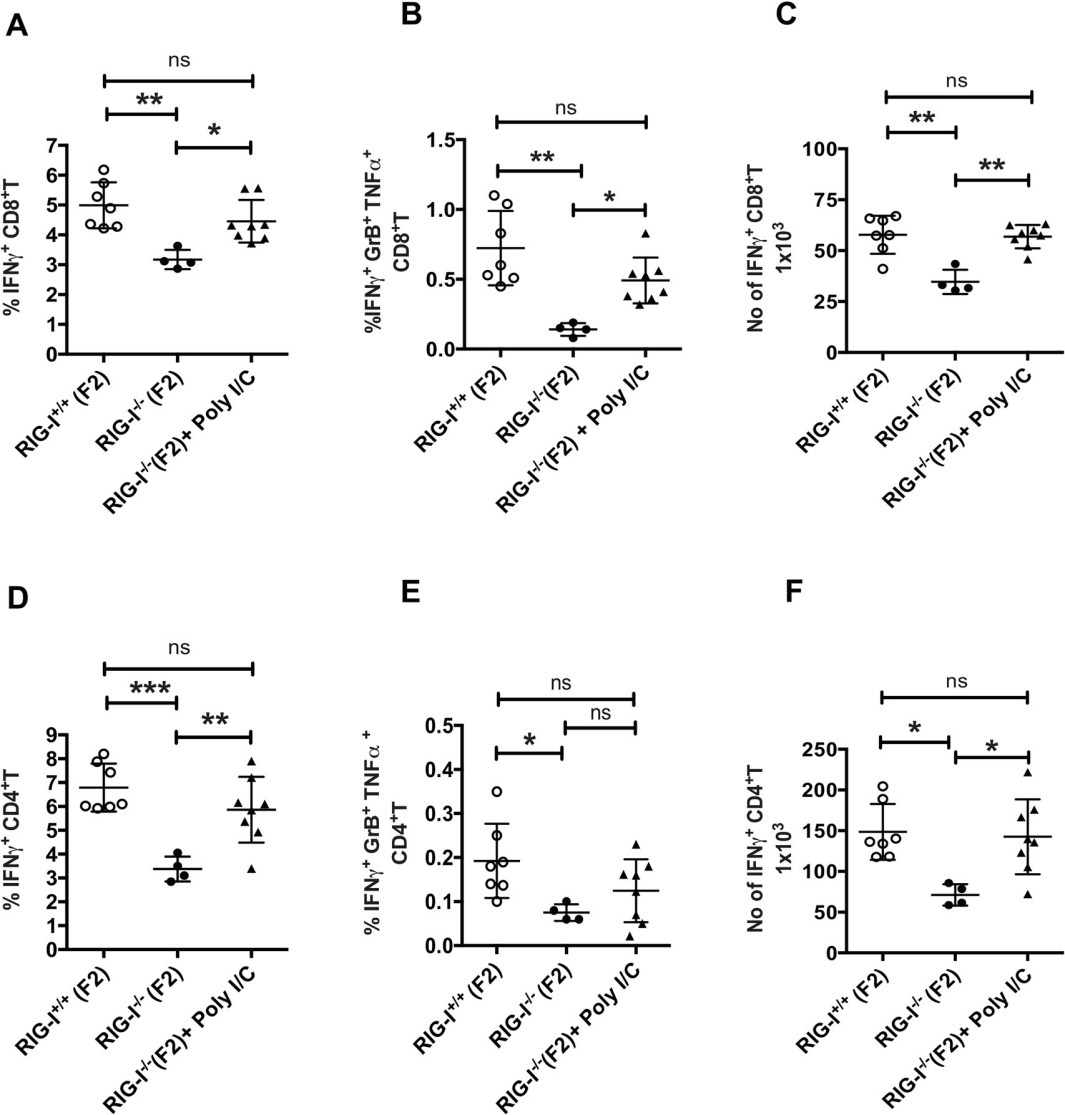
Poly I/C treatments increases T cell response in RIG-I deficient mice. RIG-I^-/-^ and RIG-I^+/+^ littermates mice were infected with PR8. At 24h, some of the RIG-I^-/-^ mice were intranasally instilled with 20μg of poly I:C. T cell responses were analyzed on day 9 post infection (A, B) Quantification of the frequencies of IFNγ or IFNγ, GrB and TNFα secreting CD8^+^ T cells. (D,E) Quantification of the frequencies of cytokine secreting CD4^+^ T cells. (C, F) Absolute number of IFNγ^+^ CD8^+^ and CD4^+^ T cells in lungs. Data shown here is a representative of two independent experiments performed with n = 7–8 mice/group. The values are expressed as mean ± SEM. * Denotes statistical significance at p<0.05 and ** denotes statistical significance at p<0.01

To further demonstrate that early activation of innate sensors is critical for efficient T cell responses, we investigated if treatment with poly I:C improves polyfunctional T cell responses in the RIG-I^-/-^ mice. Treatment with poly I:C can activate innate sensing pathways via RIG-I and MDA5. RIG-I^-/-^ mice infected with PR8 were treated with poly I:C at 24h pi and T cells responses were evaluated at day 9 pi (Fig 7). Treatment of RIG-I^-/-^ mice with poly I:C resulted in increased frequencies of IFNγ^+^ or IFNγ^+^ GrB^+^ TNFα^+^ producing T cells at levels comparable to RIG-I^+/+^ littermate controls. However, polyfunctional T cell responses were lower in control treated RIG-I^-/-^ mice as compared to other groups. Together these results demonstrate that early activation of the RIG-I-MAVS pathway is critical for efficient T cell responses against IAV.

### TLR7 deficiency affects CD4^+^ T cell responses but not CD8^+^ T cell responses against IAV

**Fig 8:**
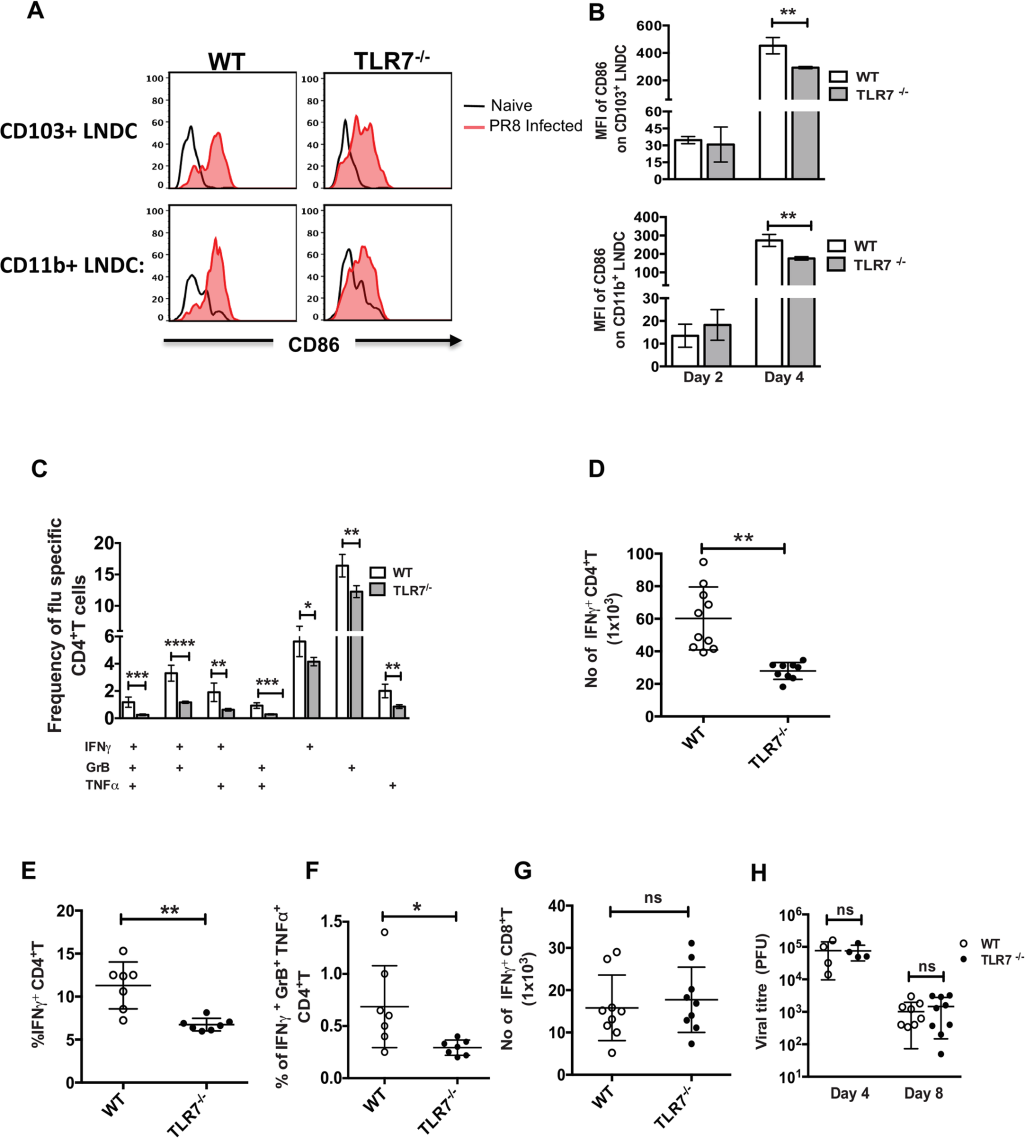
TLR7 deficient mice show impaired CD4^+^ T cell response. WT or TLR7^-/-^ mice were infected with 50PFU of PR8 and CD86 expression on migratory DC present in the mediastinal lymph node were analyzed by flow cytometry on day 2 and 4 post infection. (A) Representative histograms showing upregulation of CD86 in CD103^+^ DC (upper panel) and CD11b^+^ DC (lower panel) in MLN on day 4 post infection. (B) Quantification of MFI of CD86 upregulation on lymph node DC in infected mice with relative to naïve control. (C) Bar graphs showing the frequencies of single or polyfunctional CD4^+^T cells on day 7 post infection with PR8. (D) Absolute number of IFNγ+ CD4+T cells in lungs on day 7 post infection with PR8. (E-F) Quantification of the frequencies of IFNγ and IFNγ, GrB, TNFα secreting CD4^+^ T cells in ex vivo stimulation with class-II restricted NP peptide on day 7 post infection. (G) Absolute number of IFNγ^+^ CD8^+^T cells in lungs on day 7 post infection with PR8 (H) Viral titers in the lungs on day 4 and 8 post-infection. The limit of detection for plaque assay was 10 PFU/ml. Data shown is a presentative of two independent experiments (n = 7–11 mice/group). The values are expressed as mean ± SEM, * denotes statistical significance at p<0.05 and ** denotes statistical significance at p<0.01.

Next, we investigated the contribution of TLR7 in IAV specific T cell responses. TLR7^-/-^ and WT (C57BL/6) mice were infected with PR8 and the activation statuses of migratory DC were determined by examining for upregulation of CD86 (Fig 8A and 8B). Interestingly, we observed lowered levels of CD86 expression in both migratory DC populations in the MLN of TLR7^-/-^ mice on day 4 pi as compared to WT controls. However, the levels of CD86 expression were similar in both groups on day 2 pi. This is in contrast to results in RIG-I^-/-^ mice, where lowered CD103^+^ DC activation was observed on day 2 pi but not day 4pi. Next, we examined if the decreased expression of CD86 on LNDC affected IAV specific T cell responses by performing BMDC-T cell co-culture. Interestingly, we observed lowered frequencies of polyfunctional CD4^+^ T cells in the lungs of TLR7^-/-^ mice as compared to WT controls (Fig 8C). In addition, the absolute numbers of IAV specific CD4^+^ T cells were lower in TLR7^-/-^ as compared to WT controls (Fig 8D). Next, we evaluated CD4^+^ T cell responses against a class-II restricted IAV epitope by performing *ex-vivo* stimulation with purified peptide (Fig 8E). Again, we observed lowered frequencies of polyfunctional CD4^+^ T cells against the NP epitope in the TLR7^-/-^ group as compared to WT controls. In contrast, the polyfunctional CD8+ T cell responses, the number of IAV specific CD8^+^ T cells, and viral clearance from the lungs were unaffected in TLR7^-/-^ mice (Fig 8G and 8H and S11 Fig). These results demonstrate that TLR7 is critical for efficient CD4^+^ T cell responses but dispensable for CD8^+^ T cell responses against IAV.

## Discussion

In this study, we demonstrate that RIG-I signaling via MAVS is critical for both innate and adaptive immune responses, as we observe increased IAV infection in various cellular compartments, reduced polyfunctional T cell responses, and delayed viral clearance in RIG-I^-/-^ mice. We show that loss of RIG-I signaling leads to inefficient activation of CD103^+^ migratory DC and subsequent suboptimal priming of naïve T cells, resulting in decreased frequencies of polyfunctional T cells (Figs 4 and 6). As such, the suboptimal activation of T cells results in delayed clearance of virus and decreased protection against subsequent IAV infection from a heterologous IAV strain. (Figs 1 and 5). Exogenous addition of type I IFN to BMDC-T cell co-cultures increased expression of CD86/MHC-II in RIG-I^-/-^ BMDC and partly restored the levels of IFNγ producing CD8^+^ T cells (S10 Fig). Moreover, treatment of PR8 infected RIG-I^-/-^ mice with poly I:C at 24hpi improved polyfunctional T cell responses against IAV (Fig 7). Unlike the RIG-I-MAVS pathway, TLR7 is critical for efficient CD4+ T cell responses but dispensable for CD8+ T cell responses against IAV (Fig 8). Taken together, these results indicate that the deficiency of RIG-I and MAVS results in insufficient activation of antigen presenting cells, which functions to (1) restrict viral replication in different cellular compartments, and (2) modulate DC activation and polyfunctional T cell responses. Importantly, our study demonstrates that while the RIG-I-MAVS pathway may be sufficient for efficient CD8+ T cell responses against IAV, both the RIG-I and TLR7 pathways are required for efficient CD4+ T cell responses against IAV.

Intracellular sensors (RIG-I and NLRP3) and endosomal sensors (TLR7 and TLR3) mediate innate immune sensing of IAV infection [7,19]. Activation of these sensors leads to the production of type I IFN, which creates an antiviral state in neighboring uninfected cells, thereby restricting viral tropism. Our previous studies in IFNAR knock out mice demonstrate increased infection of both non-hematopoietic and hematopoietic cells [20]. Similarly, we observed an increased frequency of IAV infected cells from both the hematopoietic and non-hematopoietic compartments of RIG-I^-/-^ mice (S6 Fig). This demonstrates that a functional RIG-I pathway is critical for restricting IAV tropism. qPCR analysis of lung samples indicated decreased expression of IFN, ISGs, and chemokines, suggesting that early restriction of viral tropism occurs through the actions of type I IFN (S6 Fig). These data underscore the importance of early production of type I IFN, as IFNAR signaling protects different cellular populations in the lungs against IAV infection, likely through the action of antiviral ISGs such as Mx1, ISG15, PKR, and OAS. As epithelial cells and alveolar macrophages are the main targets of IAV infection as well as the major producers of type I IFN during viral infection, it is possible that the lack of RIG-I signaling in these cell types prevents production of sufficient IFN, resulting in increased IAV infection in various cellular compartments in RIG-I^-/-^ mice[21].

Apart from regulating innate immune responses through the induction of antiviral genes that directly participate in limiting viral replication, type I IFN signaling has been demonstrated to modulate adaptive immune responses [7,8,9,22,23]. To understand the role of the RIG-I-MAVS pathway in adaptive immune responses to IAV infection, we investigated if there was a delay in viral clearance in RIG-I^-/-^ and MAVS^-/-^ mice. Despite similar viral loads in the lungs of RIG-I^-/-^ and RIG-I^+/+^ mice at days 2 and 4 pi, we observed higher viral loads in the lungs in both RIG-I^-/-^ and MAVS^-/-^ mice at days 7 and 9 pi (Figs 1 and 5). This is in concurrence with previous data demonstrating no differences in viral loads in the lungs of MAVS^-/-^ and WT mice at early times of infection [13]. This delay in viral clearance suggested a defect in CD8^+^ T cell responses; thus we next examined both T cell frequency and function. Analysis of CD8^+^ T cell frequencies using tetramers showed no significant differences in the numbers of NP or PA tetramer specific CD8^+^ T cells in MAVS^-/-^ versus control mice (Fig 5), in agreement with previous studies[12,13,24,25]. However, we observed decreased frequencies of polyfunctional T cells such as IFNγ^+^ TNFα^+^ and IFNγ^+^ GrB^+^ T cells in both RIG-I^-/-^ and MAVS^-/-^ mice (Figs 2 and 5). These studies suggest that loss of RIG-I signaling not only affects the numbers of antigen specific T cells but also impairs their ability to produce multiple cytokines. For other viruses such as LCMV and WNV, the RLR pathway through MDA5 has been demonstrated to be important for efficient CD8^+^ T cell activation, as mice lacking MAVS show suboptimal CD8^+^ T cell activation [26,27]. In fact, treatment of PR8 infected RIG-I^-/-^ mice with poly I:C at 24hpi resulted in improved polyfunctional T cell responses as compared to control treated RIG-I^-/-^ mice. This is likely due to poly I:C mediated activation of the MDA5-MAVS pathway. Together, our study indicates that RIG-I signaling via MAVS is essential for determining the quality of T cell responses during IAV infection.

Type I IFN can influence the functions of DC by upregulating co-stimulatory molecules, promoting antigen processing, and increasing MHC expression [28], thereby affecting the activation of T cells. Our *ex vivo* studies in BMDC showed lowered upregulation of MHCII and CD86 expression, and suboptimal restimulation of *in vivo* primed T cells by RIG-I^-/-^ BMDC as compared to RIG-I^+/+^ BMDC (Fig 3B). Similarly, CD103^+^ migratory DC in the MLN of RIG-I^-/-^ mice showed lowered levels of CD86 expression on day 2 pi (Fig 4A). Furthermore, in our OTI adoptive transfer and Ova antigen-presentation studies, we observed lowered proliferation of CFSE labeled OTI CD8^+^ T cells in MAVS^-/-^ mice (Fig 6). These data demonstrate that lack of RIG-I signaling results in lowered activation of CD103+ migratory DC, which decreases the efficiency of T cell priming via cross-presentation. In addition, adoptive transfer experiments performed with PR8-OTI or PR8-OTII virus in MAVS^-/-^ mice also showed decreased proliferation of both OT-I and OT-II T cells, demonstrating that activation of the MAVS pathway is critical for T cell priming (Fig 6C and 6D). Interestingly, addition of exogenous type I IFN to RIG-I^-/-^ BMDC increased CD86/MHC-II expression and in part enhanced its ability to restimulate *in vivo* primed CD8+ T cells, indicating that insufficient type I IFN may be weakening migratory DC functions in RIG-I^-/-^ and MAVS^-/-^ mice (S10 Fig). Prior studies have demonstrated that IL-1β signaling in migratory CD103+ DC is critical for efficient CD8 T cell responses. As we observed decreased expression of IL-1β mRNA in the lungs of RIG-I^-/-^ mice, it is possible that decreased IL-1β may also contribute to the inefficient activation of T cell responses. Although the CD103^+^ migratory DC in the MLN of RIG-I^-/-^ mice showed lowered expression of CD86 at early times pi (day 2), we observed similar levels of CD86 expression on day 4 pi, implicating the contributions of other innate immune sensors such as TLR3 and/or TLR7 in the activation of migratory DC at later times [12,13]. In fact, our studies with TLR7^-/-^ mice indicated a lowered expression of CD86 in LNDC on day 4 pi and inefficient CD4^+^ T cell responses. This suggests a role for the RIG-I-MAVS pathway at early times and for TLR7 at later times during IAV infection. Importantly, our studies indicate that while RIG-I signaling may be sufficient for activation of CD8+ T cell responses, both RIG-I and TLR7 signaling is required for efficient CD4+ T cell responses. Prior studies indicate that IFN treatment can lead to the upregulation of TLR7 in DC. It is possible that the lowered IFNβ production in RIG-I^-/-^ mice results in decreased upregulation of TLR7 and thus indirectly affects the contribution of TLR7 during IAV infection. Further studies are necessary to determine if the RIG-I signaling in hematopoietic cells and/or non-hematopoietic cells is required for optimal activation of T cell responses. Taken together, our studies indicate that type I IFN produced early during IAV infection modulates migratory DC activation to promote efficient priming of T cells.

In summary, our study demonstrates that the RIG-I pathway acts at two levels to provide protection against IAV. The RIG-I-MAVS pathway, through the production of type I IFN and subsequent production of ISGs such as Mx1, PKR, ISG15, RNase L, etc., restricts viral infection in various cellular compartments of the lungs[7]. In addition, the cytokines produced in response to the activation of the RIG-I pathway impacts migratory DC activation and their ability to induce efficient polyfunctional T cell responses against IAV. As such, mice defective in the RIG-I-MAVS signaling pathway display delayed clearance of virus from the lungs and consequently prolonged morbidity during IAV infection. Importantly, poor T cell responses in RIG-I deficient mice during primary infection resulted in decreased protection against challenge from a heterologous IAV strain. Thus, our study demonstrates a crucial role for the RIG-I-MAVS pathway in determining the quality of T cell responses that are critical for protection against heterologous IAV strains.

## Materials and Methods

### Ethics statement

All studies were performed in accordance with the principles described by the Animal Welfare Act and the National Institutes of Health guidelines for the care and use of laboratory animals in biomedical research. The protocols for performing mice studies were reviewed and approved by Institutional Animal Care and Use committee (IACUC) at the University of Chicago and Georgia Regents University.

### Cell and viruses

MDCK cells were maintained in Minimum Essential Medium with 10% fetal bovine serum and penicillin/streptomycin (100 units/ml). IAV strains A/PR/8/1934(H1N1) (PR8, Mount Sinai strain) and X31 virus ((A/Hong Kong/1/1968) hemagglutinin and neuraminidase with remaining six segments from PR8) were grown in 10 day old embryonated eggs (Charles Rivers).

### Mice experiments

#### Strains

The generation of RIG-I^-/-^ mice has been previously described [29]. RIG-I^-/-^ mice were obtained by crossing RIG-I^+/-^ mice in 129Sv x C57BL6 background with ICR mice and the resulting RIG-I^+/-^ mice (Filial 1) were further intercrossed to get RIG-I^-/-^ (F2) mice and wild-type littermate controls RIG-I^+/+^ (F2) [5]. MAVS^-/-^ deficient mice were purchased from Jackson Laboratory and bred on site[30]. OT-I mice were originally purchased from Taconic. OT-II mice were kindly provided by Drs. Jeffrey Hubbel and Marcin Kwissa at the University of Chicago. All mice colonies were bred and housed in specific pathogen free (SPF) facilities maintained by the University of Chicago Animal Resource Centre and Georgia Regents University. All experiments were performed with gender-matched mice at 6–8 weeks of age.

#### IAV infection

Mice were anesthetized with ketamine/xylazine (i.p 80/10mg/kg) and infected by intranasal instillation of the indicated doses of virus. T cell responses were performed in mice infected with 50 PFU of PR8 virus. Body weight loss and survival experiments were performed at a dose of 100 PFU. For heterologous challenge infection, mice were first infected with 300 PFU of X31 and challenged with 1x10^6^ PFU of PR8 on day 28 post X-31 infection. In some experiments, RIG-I^-/-^ mice were intranasally administered with 20μg of Poly IC (Sigma) after 24hrs of IAV infection and T cell responses were analyzed on day 9 post infection.

### Preparation of single cell suspensions of lung and lymph nodes for flow cytometry

After euthanization, mice lungs were perfused with 10 ml of PBS and digested in 0.4mg of Collagenase in HBSS/10%FBS for 45 minutes after chopping finely with scissors. Mediastinal lymph nodes (MLN) were carefully harvested and digested in 0.2mg of collagenase in HBSS/10%FBS for 15 minutes. After digestion, lung tissues and MLN were passed through a 19G needle a few times and filtered through a 70μm cell strainer. After two washes in FACS buffer (PBS containing 1% FBS and 2mM EDTA), the cells were subjected to RBC lysis (Biowhitaker) for 3 minutes followed by two washes with FACS buffer. The single cell preparations were resuspended in blocking buffer (10μg/ml Fc receptor block in FACS buffer) and incubated for 15 minutes. For DC subset analysis, lymph node and lung cells were stained with antibodies against multiple surface antigens: anti-CD45 (2μg/ml,30-F11) (Biolegend), anti-SiglecF(1μg/ml, E50-2440) (BD Biosciences), anti-CD11c((2μg/ml, N418) (Biolegend), anti-MHC-II (2μg/ml, M5/114.15.2) (Biolegend), anti-CD103 (2μg/ml, 2E7) (eBiosciences), anti-CD11b (1μg/ml, M1/70) (Biolegend), anti-CD86 (2μg/ml, GL-1) (Biolegend), anti-Ly6G(1μg/ml, 1A8) (Biolegend), anti-Ly6C (2μg/ml HK1.4) (Biolegend), (Biolegend), anti-CD4 (2μg/ml,RM4-4) (Biolegend), anti-CD3 (2μg/ml, 145-2C11) (eBiosciences), anti-CD8 (1μg/ml, 53–6.7) (eBiosciences). IAV infected cells were identified by staining with biotinylated HA antibody (1μg/ml, PY102; Kind gift from Dr. Adolfo Garcia-Sastre) for 30 minutes on ice followed by staining with streptavidin conjugated secondary antibody (2μg/ml) (Biolegend) for 30 minutes on ice. Dead cells were stained with Live/Dead fixable near IR staining kit (Life Technologies) in PBS for 15 minutes on ice. After staining, samples were fixed with FACS FIX buffer (0.1% formaldehyde in FACS buffer) and analyzed using BD LSR-II flow cytometer at the Flow Cytometry Core Facility, The University of Chicago. Data analysis were performed using FlowJo software (Treestar Corp.). The upregulation of mean fluorescent intensity of activation markers CD86 and MHCII are represented after subtracting the MFI of CD86 or MHCII expressed in naïve or uninfected DC.

### DC migration to MLN

IAV infected mice were intranasally instilled with 50μl of 8mM CFSE (Life technologies) for labeling cells in the respiratory tract at 24hr post-infection. At 16 h post CFSE instillation, mice were euthanized and the MLN were removed and analyzed for CFSE+ migratory DC by flow cytometry.

### DC and T cell co-culture

Bone marrow derived dendritic cells (BMDC) were generated from RIG-I^+/+^ and RIG-I^-/-^ mice and T cell re-stimulation experiments were performed as previously described[31], [32]. Briefly, BMDC were first infected with PR8 at an MOI of 0.5 for 5h, washed with PBS 3 times to remove free virus, and resuspended in Iscove's Modified Dulbecco's Media complete media with FBS (IMDM, Invitrogen). For some experiments, infected BMDC were treated with IFN-I (200U/ml) before T cells were added. T cells from naïve or IAV infected RIG-I^+/+^ and RIG-I^-/-^ mice (day 7 post infection) were enriched using Pan T cells isolation kit II (Miltenyi Biotec) and co-cultured with PR8 infected BMDC at a ratio of 10:1 for 2–3 hours followed by the addition of Brefeldin A (5μg/ml; eBiosciences). DC-T cell co-cultures were further incubated for an additional 8-10hrs. The cells were first surface stained for cell surface markers, followed by intracellular staining for cytokines. For intracellular staining, cells were incubated in permeabilization and fixation buffer (BD Pharmingen) for 45 minutes followed by 2 washes in 1xPBS, 1%FBS, 0.5% Saponin (Sigma, St Louis, MO) and intracellular staining with anti-IFNγ (2μg/ml, XMG1.2) (Biolegend), anti- Granzyme B (2μg/ml, GB11) (Biolegend), anti-TNFα (2μg/ml, MP6-XT22) (eBiosciences) for 30 minutes.

### Tetramer staining

Lung lymphocytes from MAVS^-/-^ and wild-type mice were enriched by ficoll-hypaque density gradient and stained with appropriate surface antigens and PE labelled NP_366-_(ASNENMETM) and PA_224-_(SSLENFRAYV) H-2D^b^ tetramers (kindly provided by NIH tetramer facility at Emory University). Intracellular staining of IFNγ in CD8^+^T cells was also performed after *ex-vivo* stimulation with NP_366-_ peptide for 6 h in the presence of Brefeldin A.

### OT-I /OT-II T cell proliferation assay

CD8^+^ T cells or CD4^+^T cells were enriched from spleens of OT-I TCR mice or OT-II TCR mice respectively using MACS beads (Miltenyi Biotec) and labeled with 5μM of CFSE green (Molecular Probes, Invitrogen) following manufacturer’s instructions. Approximately 2x10^6^ CD8^+^ T cells or 3x10^6^ CD4^+^T cells in 200μl volume were injected intravenously followed by intranasal infection with 100PFU of PR8 or PR8 with OT-I or OT-II peptide. After 8hrs of infection, 60μg of LPS free Ovalbumin was given intranasally in PR8 infected mice. MLN were harvested on day 3 or 4 post infection for the analysis of CD8^+^T cells or CD4^+^T cells proliferation which was determined by CFSE dilution and analyzed using FlowJo software (Tree Star).

### Plaque assay

Madin-Darby Canine Kidney (MDCK) cells were seeded in 6-well dishes to be just confluent the following day. A 10-fold serial dilution of lung homogenates was prepared in 1XMEM, 0.2%BSA, and 1μg/ml TPCK-treated trypsin (Sigma). Cells were washed 2 times with PBS and incubated for 1 hour with 100μl of serially diluted tissue homogenates. After incubation, cells were washed twice in PBS and 2ml of media overlay (1x MEM with 0.2% BSA, 1μg/ml (TPCK), 0.6% agar (Oxoid), 0.1% NaHCO3; 0.01 DEAE-dextran) was added. After 48 h of incubation at 37°C, the plates were fixed with 4% formaldehyde for 1 h. Agar overlays were removed gently under running water and plaques were visualized after staining with 0.1% crystal violet solution. Plaque forming units (PFU) were counted and viral titers were expressed as PFU/ml.

### Quantitative RT-PCR

Total RNA from BMDC was extracted using Trizol (Life technologies) following the manufacturer’s instructions, and cDNA was synthesized using the Transcriptor first strand cDNA synthesis kit (Roche Diagnostics). Real-time PCR was performed on an ABI7300 real time PCR system using SYBR Green (Bioline). Primers used for qPCR analysis are listed in Supplementary table 1.

### Statistical analysis

Data were analyzed using Prism GraphPad software and statistical significance was determined by one-way ANOVA or the unpaired Student *t* test and or Fisher’s exact test. *, **, *** denote a significance of <0.05, 0.01, 0.001 respectively and Φ denotes a significance of <0.05 in Fisher’s exact test.

## Supporting Information

S1 Fig RIG-I^-/-^ mice show decreased polyfunctional T cell responses against IAV. Comparison of CD8^+^ T cell responses between RIG-I^+/+^ and RIG-I^-/-^ mice. T cells were isolated from either RIG-I^+/+^ or RIG-I^-/-^ mice on day 7 or 9 post-infection and co-cultured with infected BMDC isolated from RIG-I^+/+^ mice. The frequencies of IFNγ, TNFα and Granzyme B producing CD8^+^ T cells were analyzed by flow cytometry. (A) Representative dot plots showing polyclonal CD8^+^T cell responses on day 7 and day 9 post infection in RIG-I^+/+^ (upper panel) and RIG-I^-/-^ mice (lower panel). (B) Representative dot plots showing polyclonal CD8^+^T cell responses on day 7 in RIG-I^+/+^ (upper panel) and RIG-I^-/-^ (lower panel) littermates on day 7 post infection. The results shown are a representative of three independent experiments with similar results (n = 8–10 mice/group).
(TIF)

S2 Fig RIG-I deficient mice show impaired CD4^+^ T cell response to IAV infection. CD4 T cells from RIG-I^+/+^ or RIG-I^-/-^ mice were isolated on day 7 and 9 pi with PR8, and co-cultured with IAV infected BMDC prepared from RIG-I^+/+^ mice (A) Representative dot plots showing the frequencies of IFNγ, TNFα and Granzyme B secreting CD4+ T cells on Day or day 9 post infection. (B) Quantification for Panel A. Data shown here are a representative of two independent experiments (n = 8–10 mice/group). The values are expressed as mean ± SEM. * Denotes statistical significance at p<0.05, ** denotes statistical significance at p<0.01 and *** denotes statistical significance at p<0.001.
(TIF)

S3 Fig RIG-I deficient T cells do not display any cell intrinsic defects. (A-D) T cells were isolated from PR8 infected RIG-I^+/+^ or RIG-I^-/-^ mice and stimulated with immobilized anti-CD3/CD28 antibodies. The frequencies of T cell proliferation and upregulation of CD69 marker were monitored after the stimulation with anti CD3 and CD28 antibodies. (A, C) Frequencies of proliferating CD8^+^ and CD4^+^ T cells. (B, D) Upregulation of CD69 on CD8^+^ and CD4^+^cells. (E-F) T cells were stimulated with PMA/Ionomycin and the frequency of IFNγ producing T cells were quantified by flow cytometry. (E) CD8^+^ T cells from the lungs. (F) CD8 T cells from the MLN. Data shown here is an average of two independent experiments (n = 7 mice/group). ns denotes statistically not significant.
(TIF)

S4 Fig Quantitative RT-PCR analysis of analysis of cytokines and chemokines in BMDC upon IAV infection. Total RNA from naïve or infected BMDC was extracted and used to quantify changes in IFNβ, Mx1, ISG15, IL-1β and IL-6. Data shown here were calculated by ∆∆^CT^ method and expressed as relative fold difference from appropriate naïve controls. * denotes statistical significance at p<0.05, ** denotes statistical significance at p<0.01 and **** denotes statistical significance at p<0.0001. Data shown here is an average of two independent experiments (n = 8 mice/group).
(TIF)

S5 Fig Gating strategy for flow cytometric analysis of lung macrophages and DC subsets. Dot plots showing the flow cytometric analysis of lung DC subsets and macrophages. Dead cells were excluded from the analysis and subsequently the CD45^-^ population was gated out. CD45^+^ cells were divided into alveolar macrophages and interstitial macrophages on the basis of the expression of CD11c and Siglec F. Dendritic cells were defined as CD11c^+^ MHC-II^+^ from Siglec F^-^ cells and subsequently divided in to DC subsets on the basis of the expression of CD103 and CD11b.
(TIF)

S6 Fig Lung cellular subsets in RIG-I deficient mice show increased susceptibility to IAV infection. RIG-I^+/+^ or RIG-I^-/-^ mice were infected with 100 PFU PR8 and on day 2 and 4 post infection different cellular compartments in the lungs were analyzed for IAV infection. Infected cells were identified by staining for viral HA expression. Bar graphs showing the frequencies of HA+ cells in (A) CD45^-^ epithelial cells, (B) Alveolar macrophages, (C) Interstitial macrophages, (D) CD103^+^ lung DC, (E) CD11b^+^ lung DC. (F) qRT-PCR analysis of cytokines and chemokines in RIG-I^+/+^ and RIG-I^-/-^ mice lungs. Total RNA from the lungs was extracted at various times and used to quantify changes in IFNβ, Mx1, ISG15, CCL2, IL-1β and MIP1α. Data shown here were calculated by ∆∆^CT^ method and expressed as relative fold difference from appropriate naïve controls. Data presented here is an average of two independent experiments with total n = 9/group. * Denotes statistical significance at p<0.05 and ** denotes statistical significance at p<0.01.
(TIF)

S7 Fig Migration CD103+ and CD11b+ DC to MLN is unaffected in RIG-I deficient mice. RIG-I^+/+^ and RIG-I^-/-^ littermates were infected with 100 PFU of PR8 and instilled with 50μl of 8mM CFSE at 24hpi. After 16h, the numbers of CFSE+ labeled migratory DC present in the MLN were analyzed flow cytometry. (A) CD103+ DC and (B) CD11b+ DC. Data presented here is a representative of two independent experiments (n = 6/group).
(TIF)

S8 Fig RIG-I deficient mice show a decreased expression of CD86 and CD40 on migratory DC subsets at MLN. RIG-I^+/+^ and RIG-I^-/-^ mice were infected with 50 PFU of PR8 and CD86 expression on migratory DC present in the mediastinal lymph node and lungs were analyzed by flow cytometry. Representative offset histograms showing the expression of CD86 and CD40 in (A) CD103+ DC (B) CD11b+ DC present in the MLN and (C) CD103+ DC (D) CD11b+ DC present in the lungs. Expression in naïve (black line), day 2 (red line) and day 4 (Blue line) are shown. Data presented here is a representative of at least two independent experiments.
(TIF)

S9 Fig MAVS deficient mice display comparable IAV specific tetramer positive CD8+T cell response but show decreased polyclonal CD8^+^ T cell responses in lungs. WT or MAVS^-/-^ mice were infected with 50 PFU of PR8 and on day 7 and 9 post-infection T cell responses in the lungs were determined. (A) Representative dot plots showing the frequencies of PA (Polymerase Acidic protein) or NP (Nucleoprotein) specific CD8^+^ T cell responses in the lungs as determined using tetramers (PA_224_ and NP_366-374_). (B) Representative dot plots showing polyclonal CD8^+^ T cell responses from infected BMDC-T cell co-cultures in WT or MAVS^-/-^ mice on day 7. (C) Representative dot plots showing polyclonal CD8^+^ T cell responses from naïve BMDC/ infected BMDC—T cells co-culture in WT or MAVS^-/-^ mice on day 9. (D) Representative dot plots showing polyclonal CD8^+^ T cell responses from NP _366–374_ peptide pulsed BMDC/ T cells co-culture in WT or MAVS^-/-^ mice on day 9. Data presented here is a representative of at least three independent experiments.
(TIF)

S10 Fig Exogenous IFN restores antigen presentation and T cell activation by BMDC. CD8^+^ T cells isolated from infected RIG-I^+/+^ mice were co-cultured with BMDC generated from either RIG-I^+/+^ or RIG-I^-/-^ mice in the presence or absence of IFN I. The expression of CD86 and MHCII on DC as well as IFNγ production in CD8^+^ T cell were measured. (A) Representative histograms showing the expression of MHC-II and CD86. The expression in naïve, PR8 infected RIG-I^+/+^ and PR8 infected RIG-I^-/-^ BMDC with and without addition of exogenous IFN-I (200U/ml) are shown. (B) Quantification of MFI of CD86 and MHCII upregulation with relative to appropriate controls for panel A. (C) Quantification of IFNγ production by CD8^+^ T cells. PR8 infected RIG-I^+/+^ and RIG-I^-/-^ BMDC were treated with IFN-I or media followed by co-culturing with RIG-I^+/+^ T cells. Data shown are representative of two independent experiments (n = 8 per group). The values are expressed as mean ± SEM. * Denotes statistical significance at p<0.05 and ** denotes statistical significance at p<0.01
(TIF)

S11 Fig TLR7 deficient mice display comparable IAV specific tetramer positive and polyclonal CD8^+^ T cell responses in lungs. WT or TLR7^-/-^ mice were infected with 50 PFU of PR8 and on day 7 post-infection T cell responses and viral titers in the lungs were determined. (A) Quantification of the frequency of PA and NP specific lung CD8^+^ T cells as determined using tetramers (PA_224_ and NP_366-374_) on day 7. (B) Bar graph showing the frequencies of single or polyfunctional CD8^+^T cells on day 7 post infection with PR8. Data shown are representative of two independent experiments (n = 8–11 per group)
(TIF)
