# Supplementary figures and images for: RIG-I Signaling Is Critical for Efficient Polyfunctional T Cell Responses during Influenza Virus Infection
Source: PLoS Pathog. 2016 Jul 20;12(7):e1005754. doi: 10.1371/journal.ppat.1005754 (PMC4954706; doi:10.1371/journal.ppat.1005754)

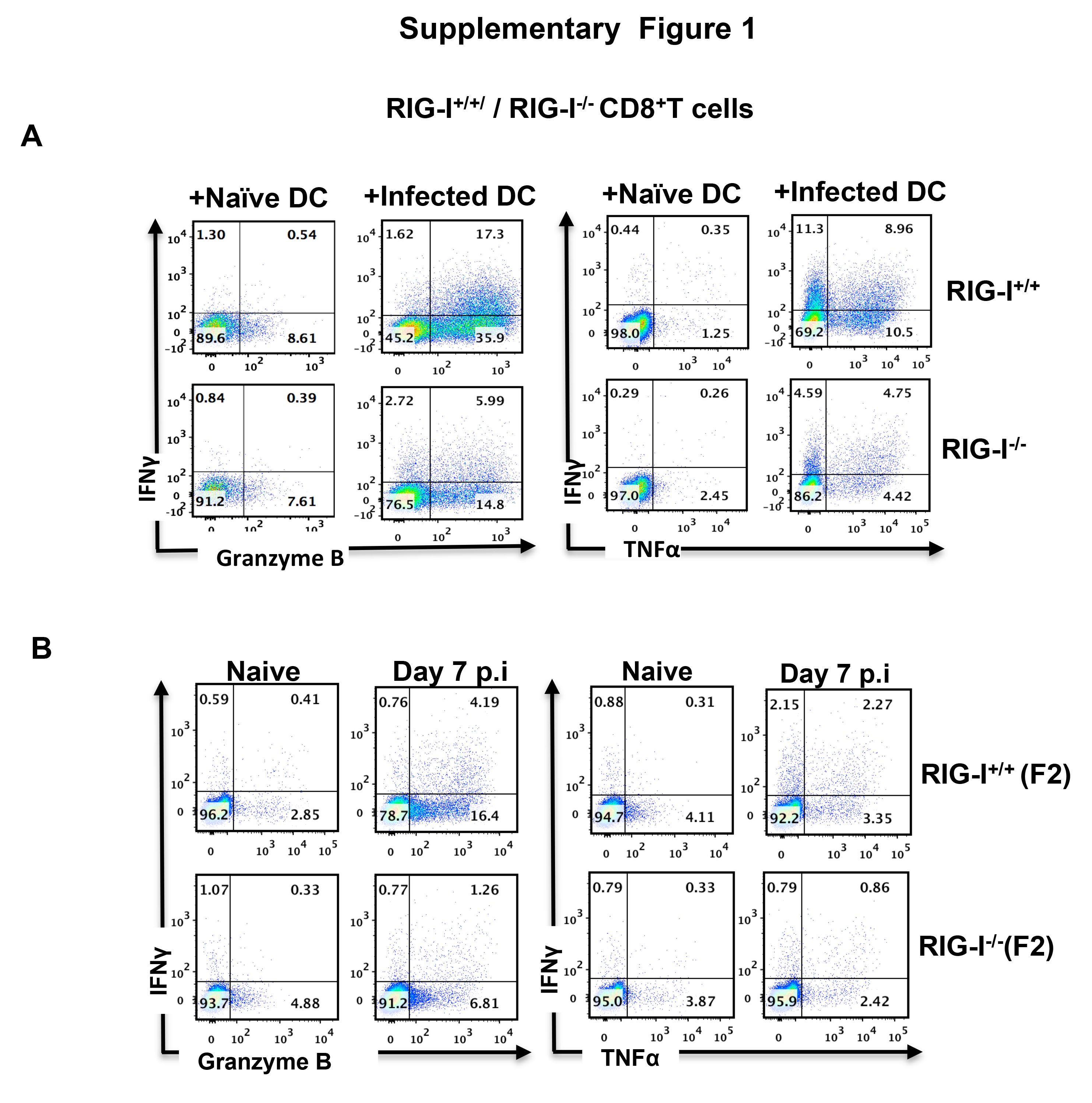

Supplement: S1 Fig — Comparison of CD8+ T cell responses between RIG-I+/+ and RIG-I-/- mice. T cells were isolated from either RIG-I+/+ or RIG-I-/- mice on day 7 or 9 post-infection and co-cultured with infected BMDC isolated from RIG-I+/+ mice. The frequencies of IFNγ, TNFα and Granzyme B producing CD8+ T cells were analyzed by flow cytometry. (A) Representative dot plots showing polyclonal CD8+T cell responses on day 7 and day 9 post infection in RIG-I+/+ (upper panel) and RIG-I-/- mice (lower panel). (B) Representative dot plots showing polyclonal CD8+T cell responses on day 7 in RIG-I+/+ (upper panel) and RIG-I-/- (lower panel) littermates on day 7 post infection. The results shown are a representative of three independent experiments with similar results (n = 8–10 mice/group). (TIF) [file ppat.1005754.s001.tif]

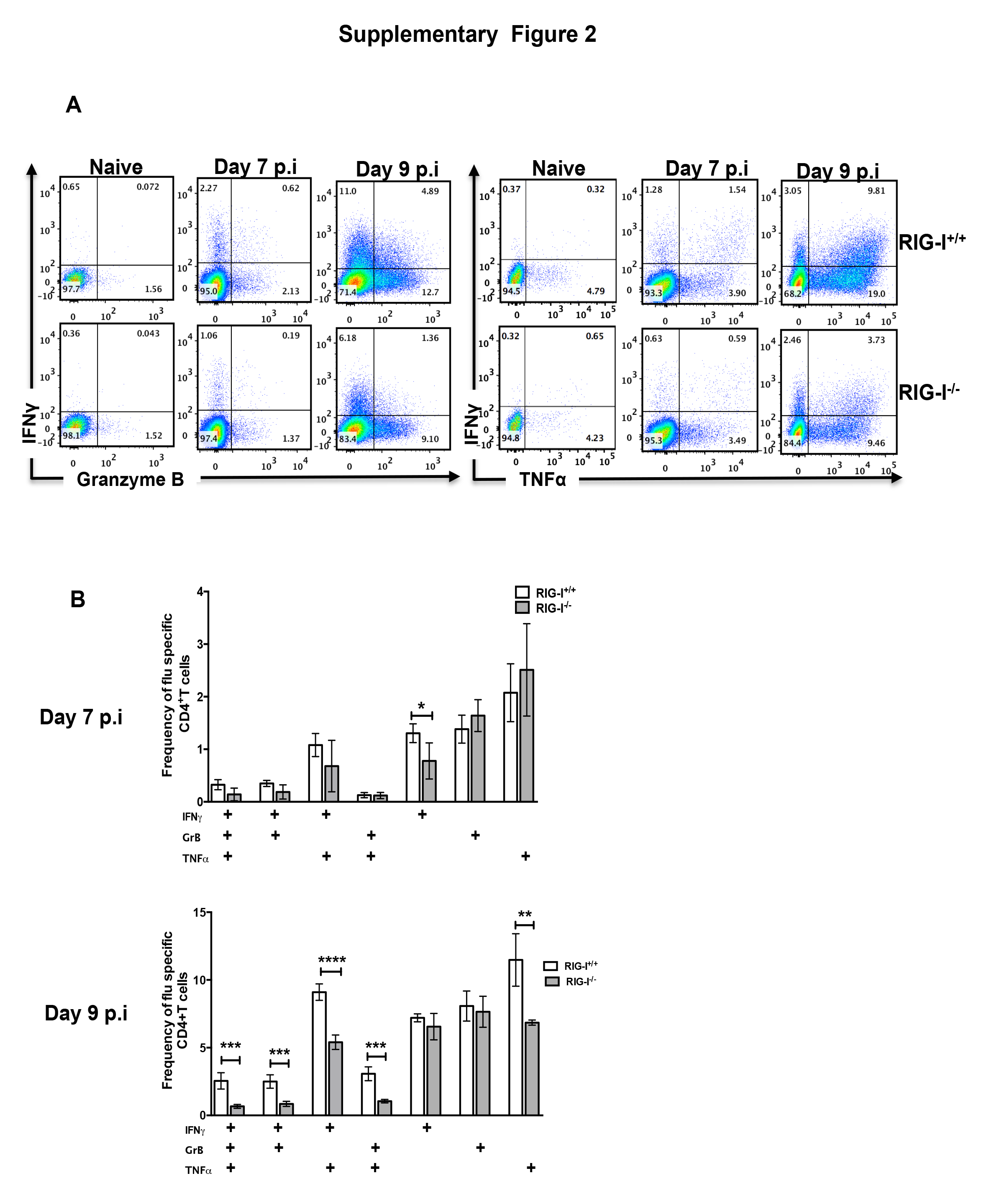

Supplement: S2 Fig — CD4 T cells from RIG-I+/+ or RIG-I-/- mice were isolated on day 7 and 9 pi with PR8, and co-cultured with IAV infected BMDC prepared from RIG-I+/+ mice (A) Representative dot plots showing the frequencies of IFNγ, TNFα and Granzyme B secreting CD4+ T cells on Day or day 9 post infection. (B) Quantification for Panel A. Data shown here are a representative of two independent experiments (n = 8–10 mice/group). The values are expressed as mean ± SEM. * Denotes statistical significance at p<0.05, ** denotes statistical significance at p<0.01 and *** denotes statistical significance at p<0.001. (TIF) [file ppat.1005754.s002.tif]

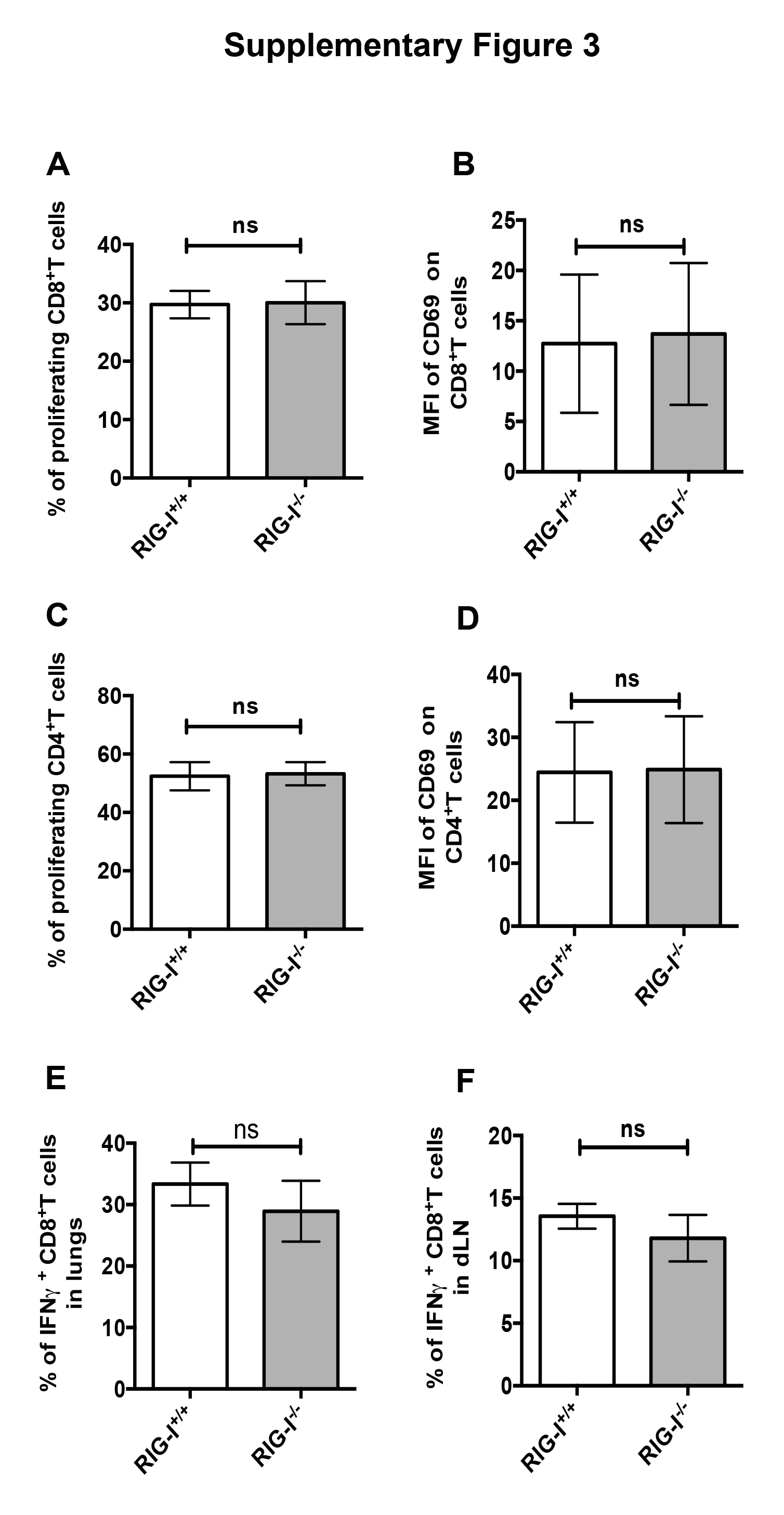

Supplement: S3 Fig — (A-D) T cells were isolated from PR8 infected RIG-I+/+ or RIG-I-/- mice and stimulated with immobilized anti-CD3/CD28 antibodies. The frequencies of T cell proliferation and upregulation of CD69 marker were monitored after the stimulation with anti CD3 and CD28 antibodies. (A, C) Frequencies of proliferating CD8+ and CD4+ T cells. (B, D) Upregulation of CD69 on CD8+ and CD4+cells. (E-F) T cells were stimulated with PMA/Ionomycin and the frequency of IFNγ producing T cells were quantified by flow cytometry. (E) CD8+ T cells from the lungs. (F) CD8 T cells from the MLN. Data shown here is an average of two independent experiments (n = 7 mice/group). ns denotes statistically not significant. (TIF) [file ppat.1005754.s003.tif]

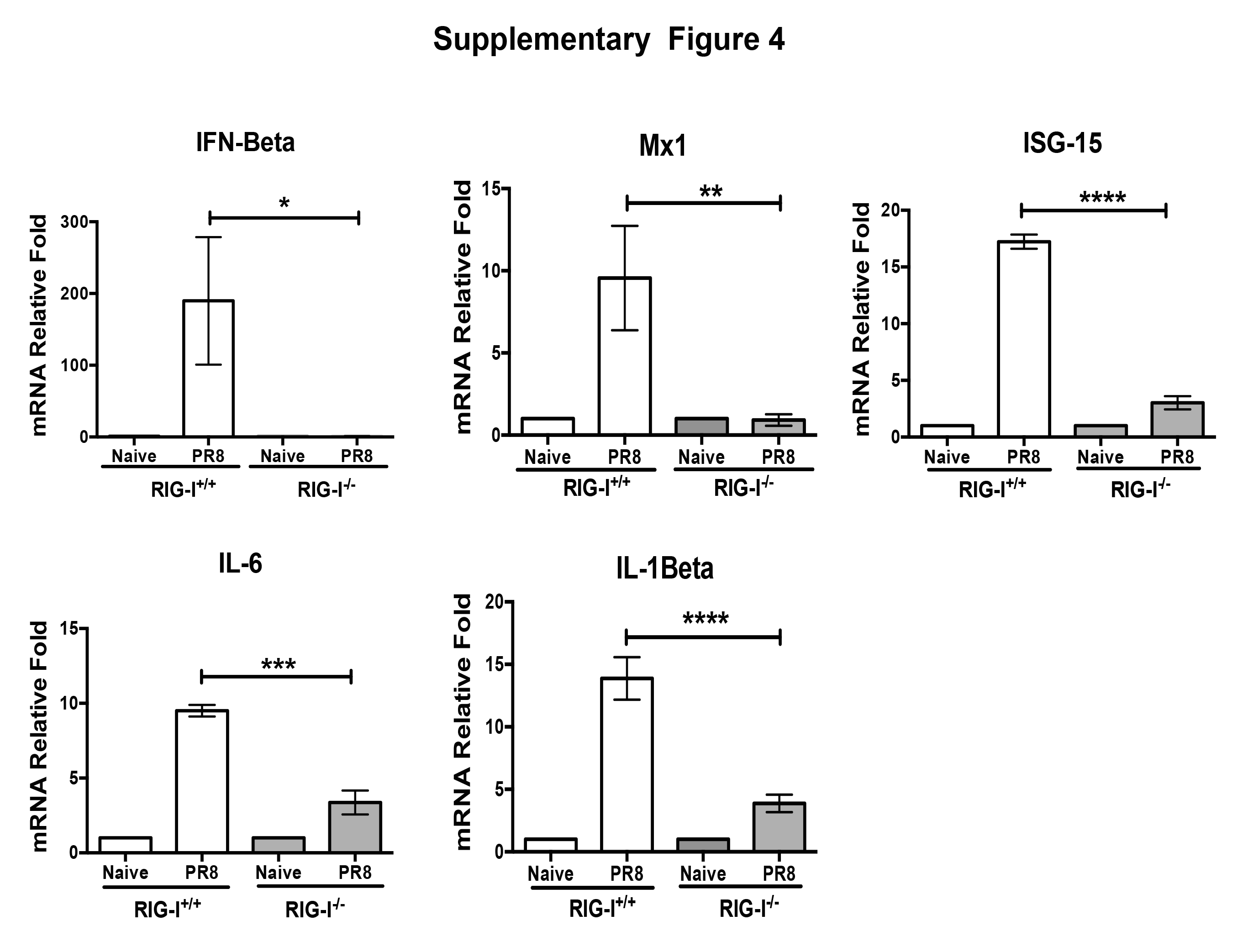

Supplement: S4 Fig — Total RNA from naïve or infected BMDC was extracted and used to quantify changes in IFNβ, Mx1, ISG15, IL-1β and IL-6. Data shown here were calculated by ∆∆CT method and expressed as relative fold difference from appropriate naïve controls. * denotes statistical significance at p<0.05, ** denotes statistical significance at p<0.01 and **** denotes statistical significance at p<0.0001. Data shown here is an average of two independent experiments (n = 8 mice/group). (TIF) [file ppat.1005754.s004.tif]

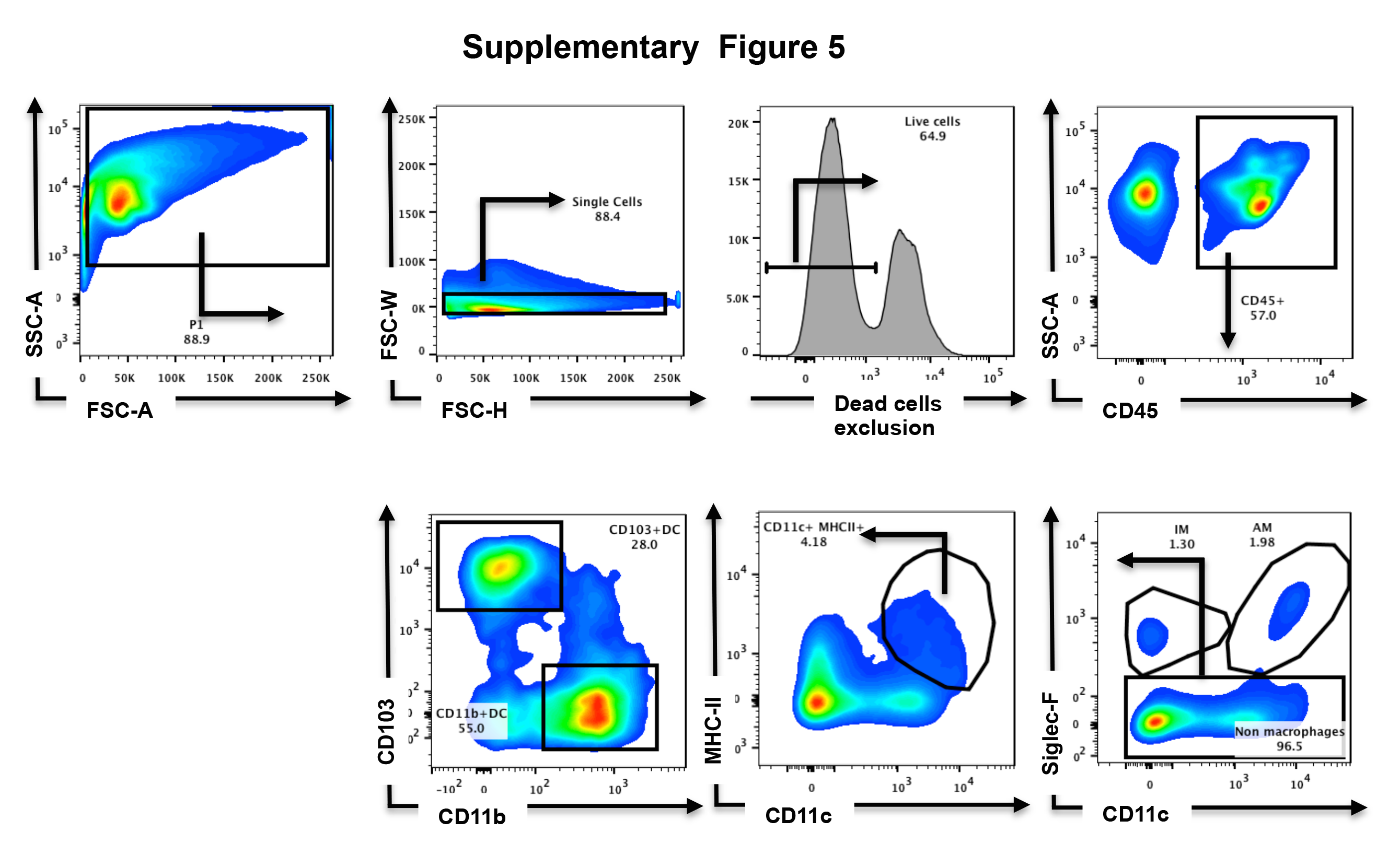

Supplement: S5 Fig — Dot plots showing the flow cytometric analysis of lung DC subsets and macrophages. Dead cells were excluded from the analysis and subsequently the CD45- population was gated out. CD45+ cells were divided into alveolar macrophages and interstitial macrophages on the basis of the expression of CD11c and Siglec F. Dendritic cells were defined as CD11c+ MHC-II+ from Siglec F- cells and subsequently divided in to DC subsets on the basis of the expression of CD103 and CD11b. (TIF) [file ppat.1005754.s005.tif]

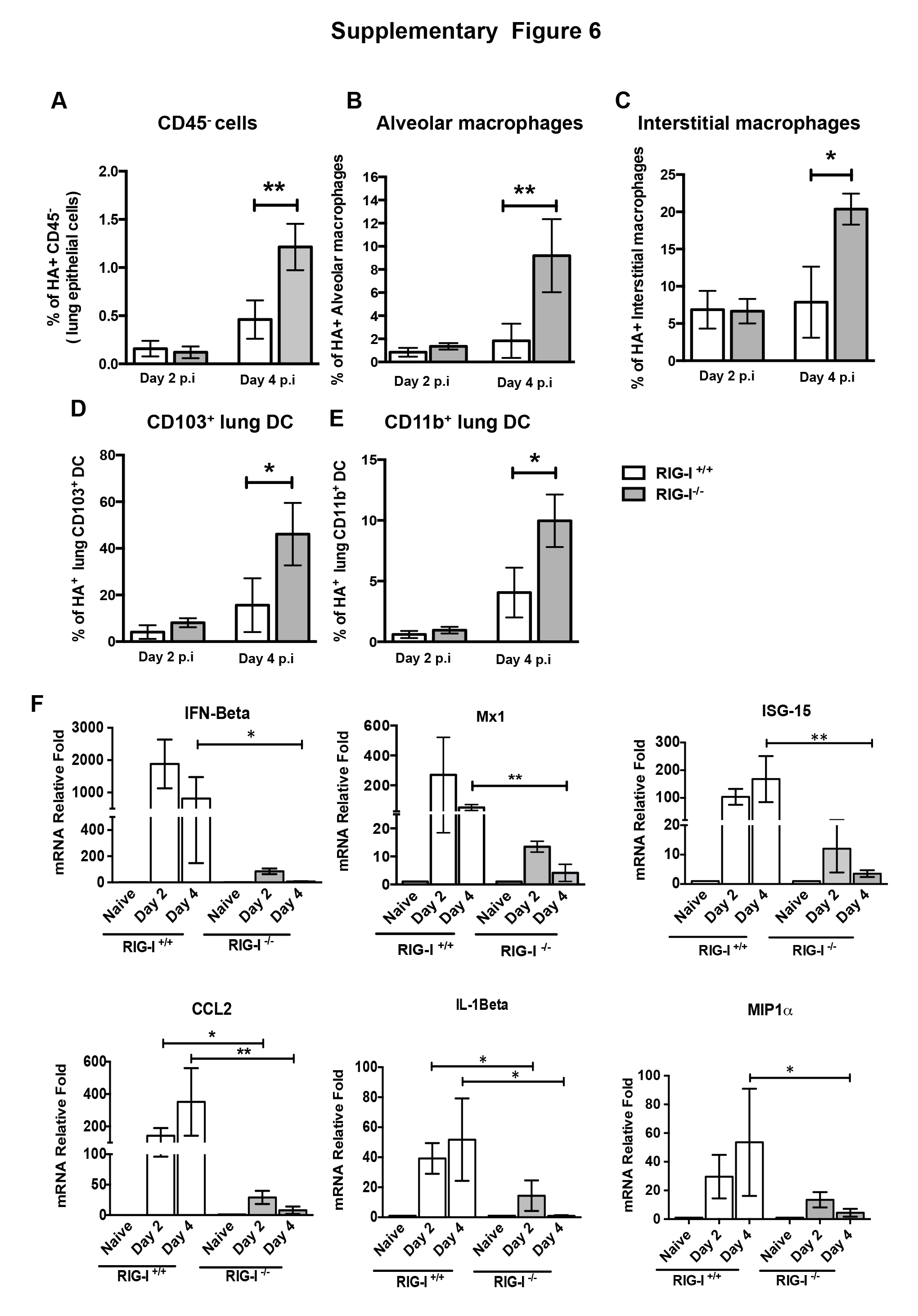

Supplement: S6 Fig — RIG-I+/+ or RIG-I-/- mice were infected with 100 PFU PR8 and on day 2 and 4 post infection different cellular compartments in the lungs were analyzed for IAV infection. Infected cells were identified by staining for viral HA expression. Bar graphs showing the frequencies of HA+ cells in (A) CD45- epithelial cells, (B) Alveolar macrophages, (C) Interstitial macrophages, (D) CD103+ lung DC, (E) CD11b+ lung DC. (F) qRT-PCR analysis of cytokines and chemokines in RIG-I+/+ and RIG-I-/- mice lungs. Total RNA from the lungs was extracted at various times and used to quantify changes in IFNβ, Mx1, ISG15, CCL2, IL-1β and MIP1α. Data shown here were calculated by ∆∆CT method and expressed as relative fold difference from appropriate naïve controls. Data presented here is an average of two independent experiments with total n = 9/group. * Denotes statistical significance at p<0.05 and ** denotes statistical significance at p<0.01. (TIF) [file ppat.1005754.s006.tif]

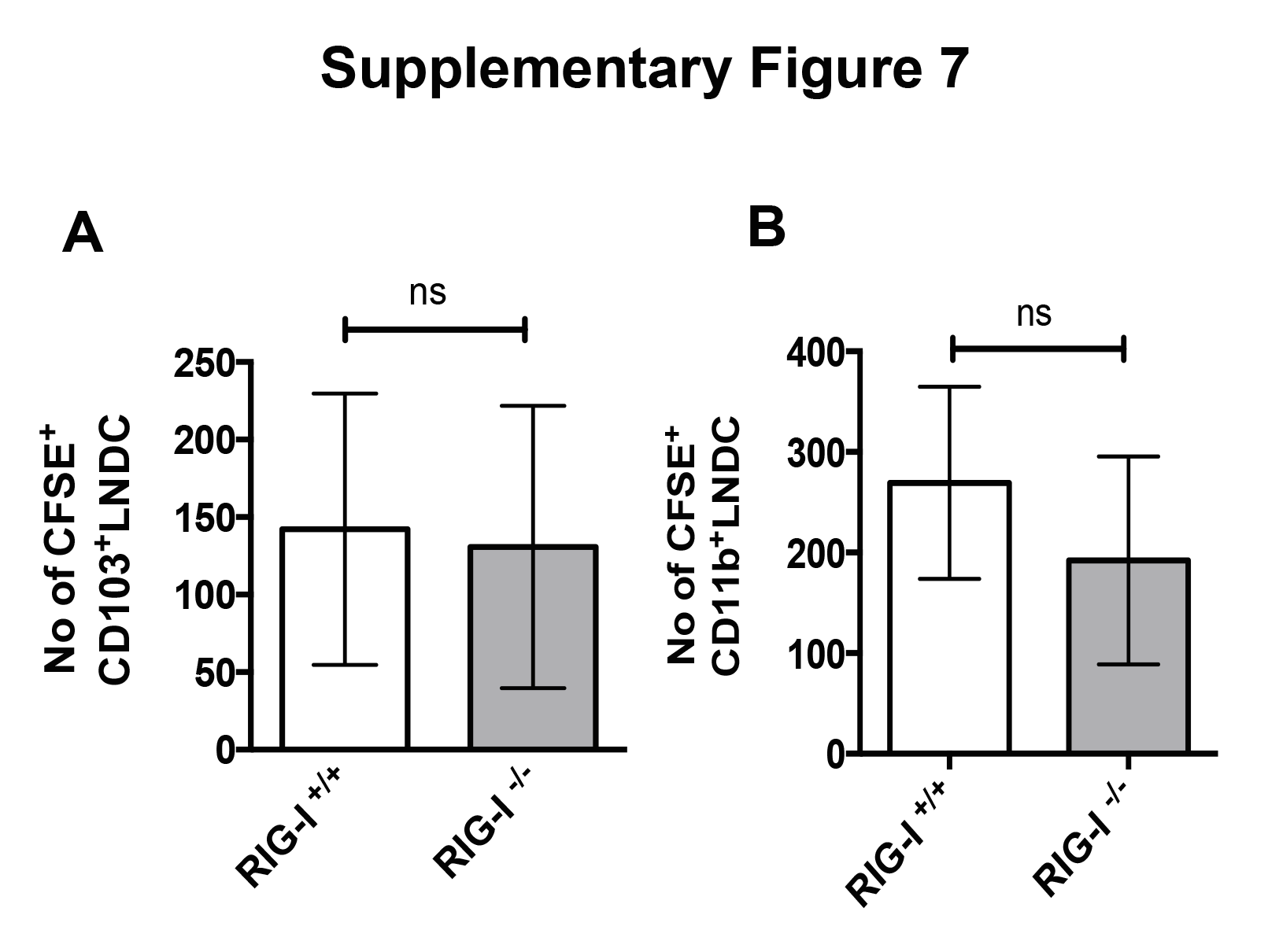

Supplement: S7 Fig — RIG-I+/+ and RIG-I-/- littermates were infected with 100 PFU of PR8 and instilled with 50μl of 8mM CFSE at 24hpi. After 16h, the numbers of CFSE+ labeled migratory DC present in the MLN were analyzed flow cytometry. (A) CD103+ DC and (B) CD11b+ DC. Data presented here is a representative of two independent experiments (n = 6/group). (TIF) [file ppat.1005754.s007.tif]

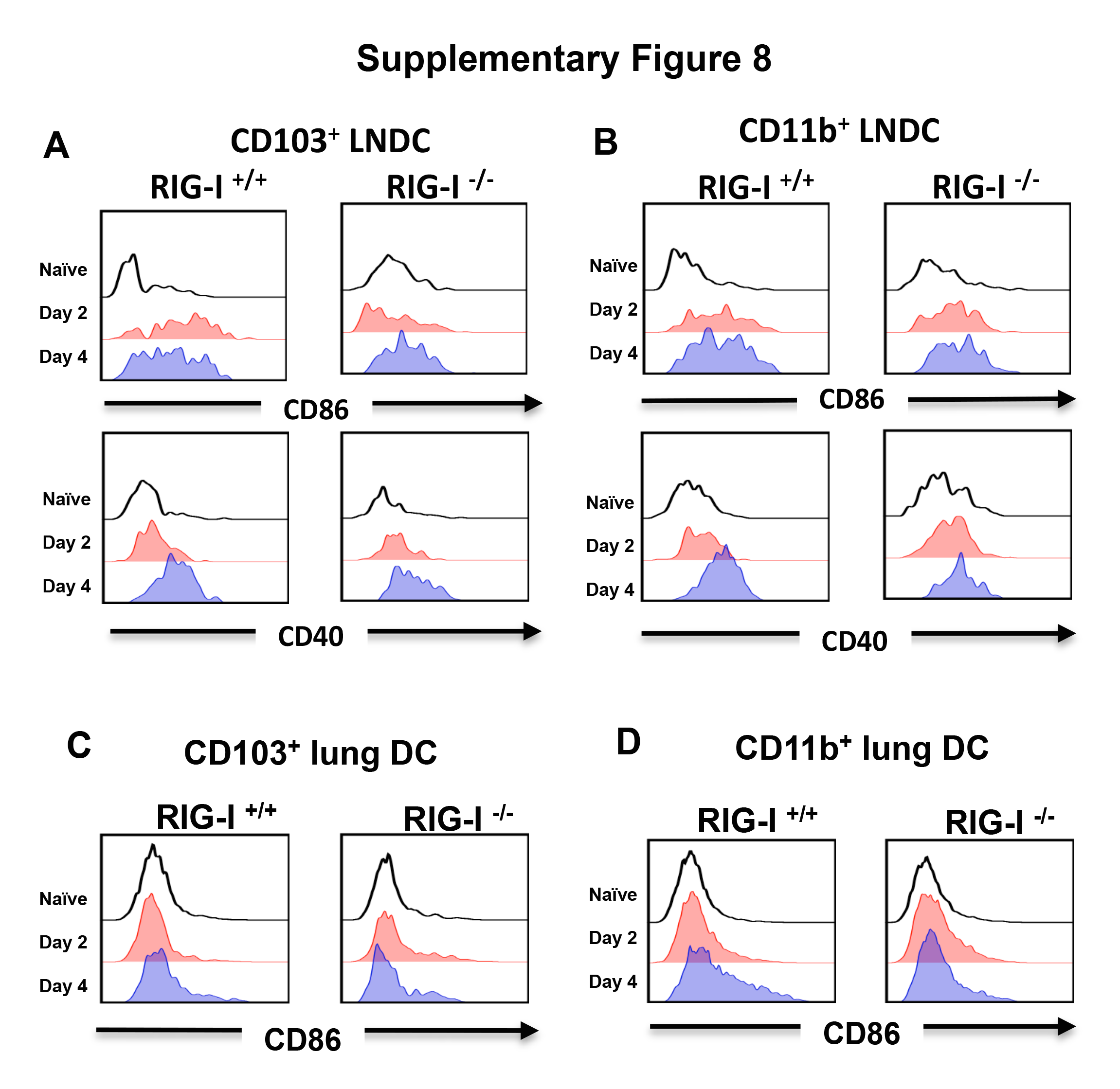

Supplement: S8 Fig — RIG-I+/+ and RIG-I-/- mice were infected with 50 PFU of PR8 and CD86 expression on migratory DC present in the mediastinal lymph node and lungs were analyzed by flow cytometry. Representative offset histograms showing the expression of CD86 and CD40 in (A) CD103+ DC (B) CD11b+ DC present in the MLN and (C) CD103+ DC (D) CD11b+ DC present in the lungs. Expression in naïve (black line), day 2 (red line) and day 4 (Blue line) are shown. Data presented here is a representative of at least two independent experiments. (TIF) [file ppat.1005754.s008.tif]

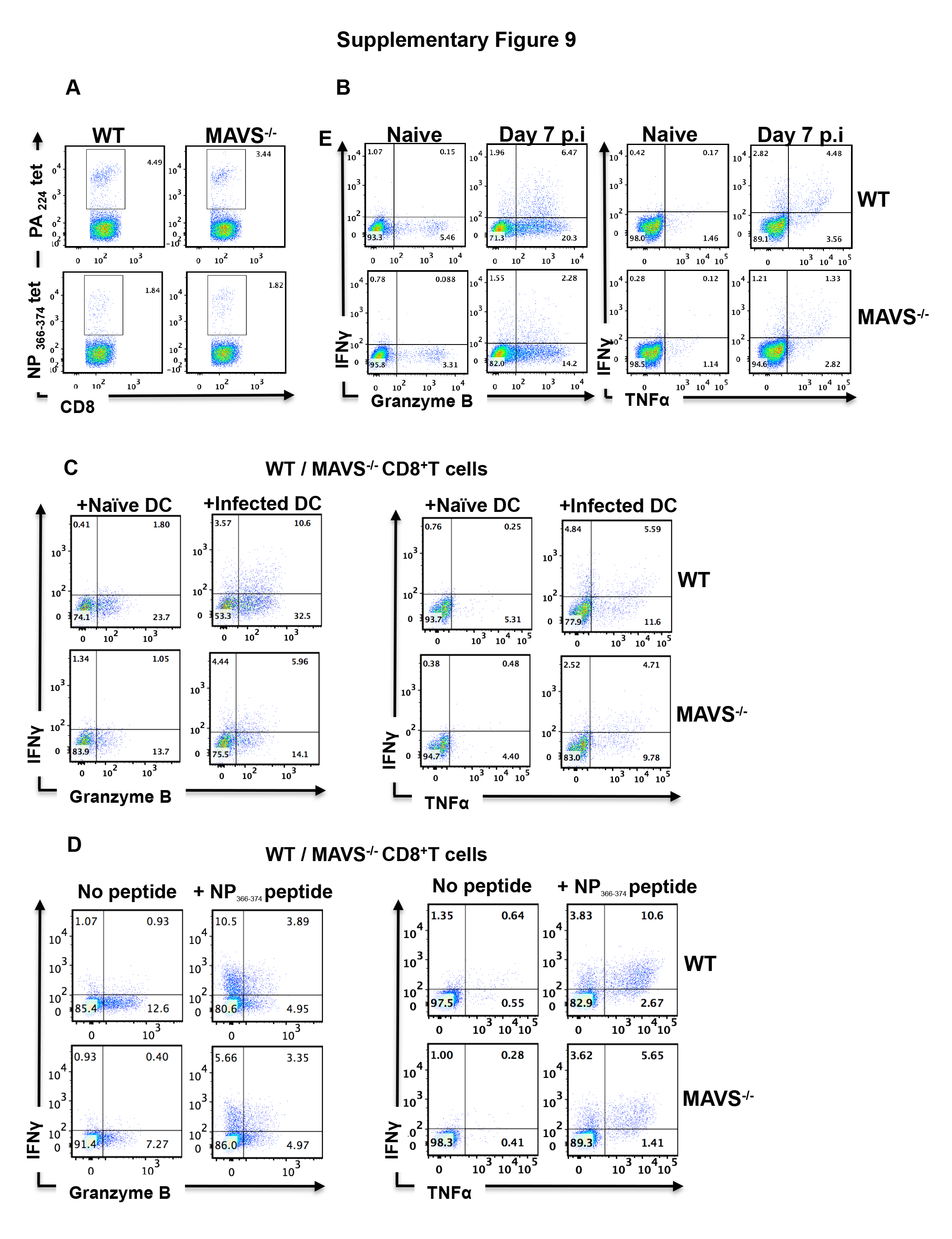

Supplement: S9 Fig — WT or MAVS-/- mice were infected with 50 PFU of PR8 and on day 7 and 9 post-infection T cell responses in the lungs were determined. (A) Representative dot plots showing the frequencies of PA (Polymerase Acidic protein) or NP (Nucleoprotein) specific CD8+ T cell responses in the lungs as determined using tetramers (PA224 and NP366-374). (B) Representative dot plots showing polyclonal CD8+ T cell responses from infected BMDC-T cell co-cultures in WT or MAVS-/- mice on day 7. (C) Representative dot plots showing polyclonal CD8+ T cell responses from naïve BMDC/ infected BMDC—T cells co-culture in WT or MAVS-/- mice on day 9. (D) Representative dot plots showing polyclonal CD8+ T cell responses from NP 366–374 peptide pulsed BMDC/ T cells co-culture in WT or MAVS-/- mice on day 9. Data presented here is a representative of at least three independent experiments. (TIF) [file ppat.1005754.s009.tif]

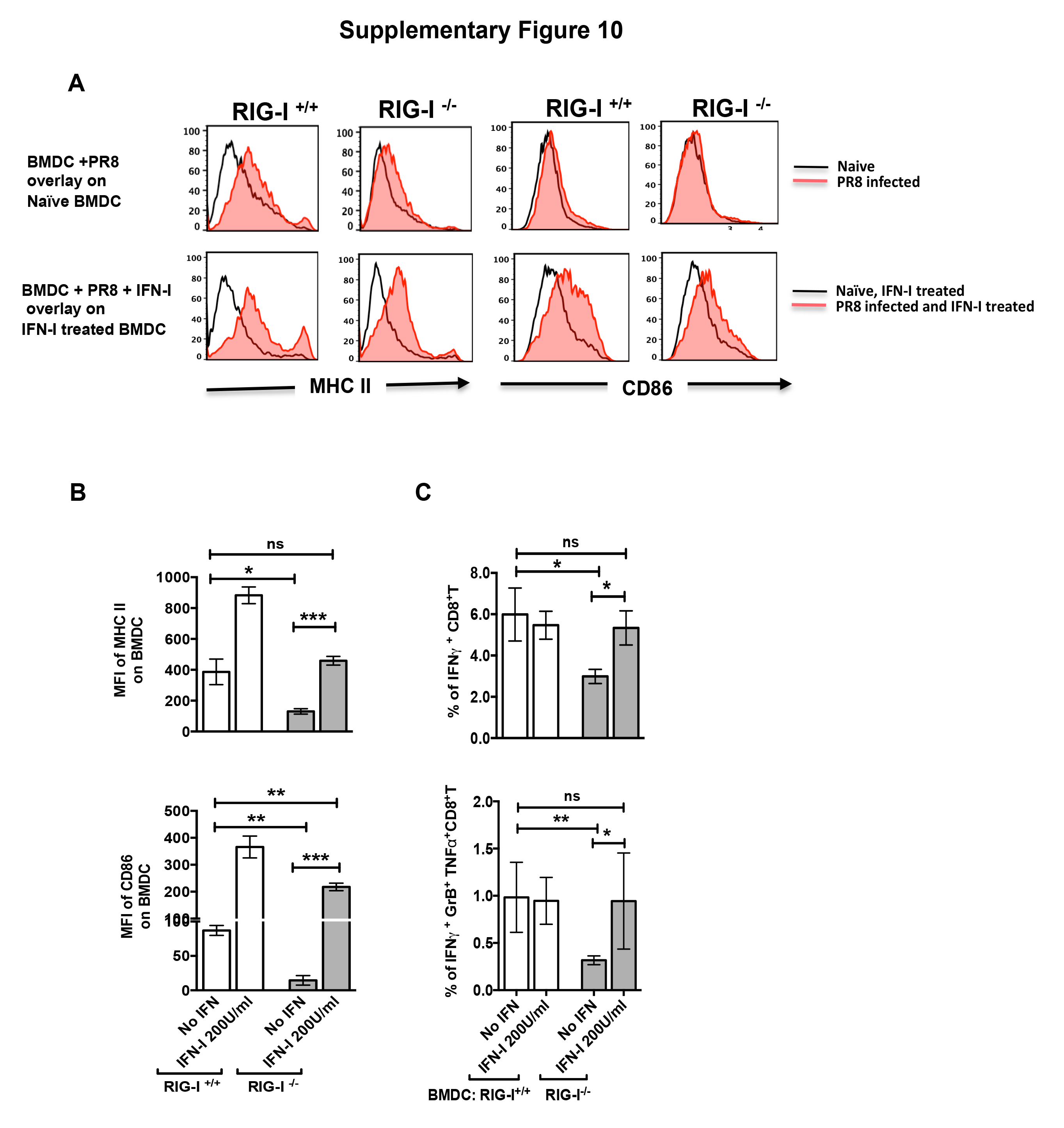

Supplement: S10 Fig — CD8+ T cells isolated from infected RIG-I+/+ mice were co-cultured with BMDC generated from either RIG-I+/+ or RIG-I-/- mice in the presence or absence of IFN I. The expression of CD86 and MHCII on DC as well as IFNγ production in CD8+ T cell were measured. (A) Representative histograms showing the expression of MHC-II and CD86. The expression in naïve, PR8 infected RIG-I+/+ and PR8 infected RIG-I-/- BMDC with and without addition of exogenous IFN-I (200U/ml) are shown. (B) Quantification of MFI of CD86 and MHCII upregulation with relative to appropriate controls for panel A. (C) Quantification of IFNγ production by CD8+ T cells. PR8 infected RIG-I+/+ and RIG-I-/- BMDC were treated with IFN-I or media followed by co-culturing with RIG-I+/+ T cells. Data shown are representative of two independent experiments (n = 8 per group). The values are expressed as mean ± SEM. * Denotes statistical significance at p<0.05 and ** denotes statistical significance at p<0.01 (TIF) [file ppat.1005754.s010.tif]

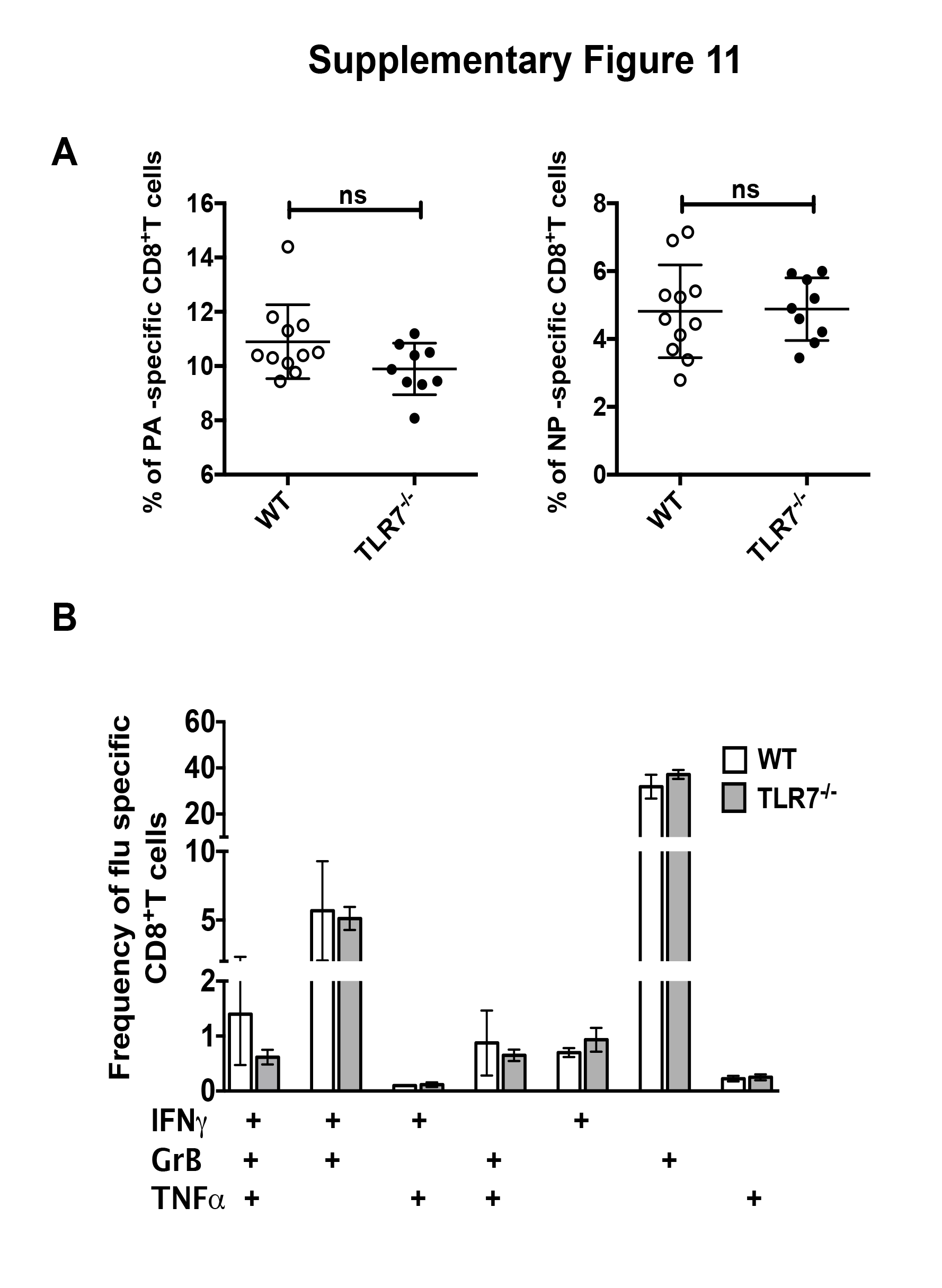

Supplement: S11 Fig — WT or TLR7-/- mice were infected with 50 PFU of PR8 and on day 7 post-infection T cell responses and viral titers in the lungs were determined. (A) Quantification of the frequency of PA and NP specific lung CD8+ T cells as determined using tetramers (PA224 and NP366-374) on day 7. (B) Bar graph showing the frequencies of single or polyfunctional CD8+T cells on day 7 post infection with PR8. Data shown are representative of two independent experiments (n = 8–11 per group) (TIF) [file ppat.1005754.s011.tif]
